# *DCLK1* Variants Are Associated across Schizophrenia and Attention Deficit/Hyperactivity Disorder

**DOI:** 10.1371/journal.pone.0035424

**Published:** 2012-04-23

**Authors:** Bjarte Håvik, Franziska A. Degenhardt, Stefan Johansson, Carla P. D. Fernandes, Anke Hinney, André Scherag, Helle Lybæk, Srdjan Djurovic, Andrea Christoforou, Kari M. Ersland, Sudheer Giddaluru, Michael C. O'Donovan, Michael J. Owen, Nick Craddock, Thomas W. Mühleisen, Manuel Mattheisen, Benno G. Schimmelmann, Tobias Renner, Andreas Warnke, Beate Herpertz-Dahlmann, Judith Sinzig, Özgür Albayrak, Marcella Rietschel, Markus M. Nöthen, Clive R. Bramham, Thomas Werge, Johannes Hebebrand, Jan Haavik, Ole A. Andreassen, Sven Cichon, Vidar M. Steen, Stéphanie Le Hellard

**Affiliations:** 1 Dr. E. Martens Research Group for Biological Psychiatry, Department of Clinical Medicine, University of Bergen, Bergen, Norway; 2 Center for Medical Genetics and Molecular Medicine, Haukeland University Hospital, Bergen, Norway; 3 Department of Genomics, Life & Brain Center, University of Bonn, Bonn, Germany; 4 Institute of Human Genetics, University of Bonn, Bonn, Germany; 5 Department of Biomedicine, University of Bergen, Bergen, Norway; 6 Department of Child and Adolescent Psychiatry, University of Duisburg-Essen, Essen, Germany; 7 Institute for Medical Informatics, Biometry and Epidemiology, University of Duisburg-Essen, Essen, Germany; 8 Institute of Psychiatry, University of Oslo, Oslo, Norway; 9 Department of Psychological Medicine and Neurology, MRC Centre for Neuropsychiatric Genetics and Genomics, Neuroscience and Mental Health Research Institute, Cardiff University, Cardiff, United Kingdom; 10 Institute for Medical Biometry, Informatics, and Epidemiology (IMBIE), University of Bonn, Bonn, Germany; 11 University Hospital of Child and Adolescent Psychiatry Bern, Bern, Switzerland; 12 Department of Child and Adolescent Psychiatry, Julius-Maximilians-University Würzburg, Würzburg, Germany; 13 Department of Child and Adolescent Psychiatry, Psychosomatics and Psychotherapy, University Clinics, Technical University Aachen, Aachen, Germany; 14 Department for Child and Adolescent Psychiatry and Psychotherapy, University of Cologne, Koln, Germany; 15 Department for Child and Adolescent Psychiatry and Psychotherapy, LVR- Klinik Bonn, Bonn, Germany; 16 Department of Psychiatry, University of Bonn, Bonn, Germany; 17 German Centre for Neurodegenerative Disorders (DZNE), Bonn, Germany; 18 Research Institute of Biological Psychiatry, Copenhagen University Hospital, Roskilde, Denmark; 19 Division of Psychiatry, Haukeland University Hospital, Bergen, Norway; 20 Division of Mental Health and Addiction, Institute of Clinical Medicine, University of Oslo, Oslo University Hospital, Oslo, Norway; 21 Institute of Neuroscience and Medicine (INM-1), Research Center Juelich, Juelich, Germany; Chiba University Center for Forensic Mental Health, Japan

## Abstract

Doublecortin and calmodulin like kinase 1 (DCLK1) is implicated in synaptic plasticity and neurodevelopment. Genetic variants in *DCLK1* are associated with cognitive traits, specifically verbal memory and general cognition. We investigated the role of *DCLK1* variants in three psychiatric disorders that have neuro-cognitive dysfunctions: schizophrenia (SCZ), bipolar affective disorder (BP) and attention deficit/hyperactivity disorder (ADHD). We mined six genome wide association studies (GWASs) that were available publically or through collaboration; three for BP, two for SCZ and one for ADHD. We also genotyped the *DCLK1* region in additional samples of cases with SCZ, BP or ADHD and controls that had not been whole-genome typed. In total, 9895 subjects were analysed, including 5308 normal controls and 4,587 patients (1,125 with SCZ, 2,496 with BP and 966 with ADHD). Several *DCLK1* variants were associated with disease phenotypes in the different samples. The main effect was observed for rs7989807 in intron 3, which was strongly associated with SCZ alone and even more so when cases with SCZ and ADHD were combined (P-value = 4×10^−5^ and 4×10^−6^, respectively). Associations were also observed with additional markers in intron 3 (combination of SCZ, ADHD and BP), intron 19 (SCZ+BP) and the 3′UTR (SCZ+BP). Our results suggest that genetic variants in *DCLK1* are associated with SCZ and, to a lesser extent, with ADHD and BP. Interestingly the association is strongest when SCZ and ADHD are considered together, suggesting common genetic susceptibility. Given that *DCLK1* variants were previously found to be associated with cognitive traits, these results are consistent with the role of DCLK1 in neurodevelopment and synaptic plasticity.

## Introduction

Neuropsychological impairments are core symptoms of several psychiatric disorders like schizophrenia (SCZ) [1], [2], [3], attention-deficit/hyperactivity disorder (ADHD) [4], [5], and bipolar affective disorder (BP) [6], [7]. Although genetic factors play a major role in psychiatric disorders, only a few genes implicated in these conditions have been identified, probably due, at least in part, to the difficulty of identifying reliable phenotypes. It has been suggested that the chances of identifying the genes underlying these psychiatric disorders would be increased by studying clearly defined endophenotypes [8] or intermediate phenotypes [7], [8], [9]. Several highly heritable neuro-cognitive traits have been proposed as relevant endophenotypes, and a number of genes have been identified that show association with these traits *per se* as well as with related psychiatric disorders [10], [11], [12], [13]. The common associations across psychiatric phenotypes and relevant neuropsychological traits could reflect a general effect of these genes on specific neuronal functions. For instance, the potential etiological role of the *brain derived neurotrophic factor* (*BDNF*) gene in psychiatric disorders and cognitive traits could reflect its central role in synaptic plasticity [14]. We hypothesised that other genes functionally related to BDNF could also be implicated in cognition and psychiatric disorders. We previously carried out a gene expression analysis of BDNF-induced long-term potentiation (LTP) of synaptic transmission in the hippocampus of live rats [15]. We identified a set of seven genes that were up-regulated during this treatment and were confirmed to be up-regulated in another model of synaptic plasticity [15]. We then investigated whether genetic variants from this set of “BDNF up-regulated” genes were implicated in cognitive traits. We showed that variants in one of the seven genes, *DCLK1* (*doublecortin and calmodulin like kinase 1*), were significantly associated with verbal memory and IQ scores in three independent samples of healthy adults from Norway and Scotland [16].

*DCLK1* (previously known as *DCAMKL1*) is a complex gene that is translated into at least 10 proteins with two major classes of transcripts. The long variants contain exons 1 to 20 (except for exons 6 and 8), while the short variants contain exons 6 to 20 (except for exon 8) [17]. Two other variants are also found: the Ca(2+)/calmodulin dependent protein kinase (CaMK)-related peptide (exons 6 to 8; also known as CARP), and the doublecortin-like variant (exons 1–5, 7 and 8). In rodents, differential expression has been described, with long variants expressed during embryogenesis and short variants in adulthood [17]. In humans, this contrast is less pronounced; long variants are more strongly expressed in embryos, while short variants are predominant in adults, but all variants are seen throughout the life span [16]. In man, the *DCLK1* gene is highly expressed in the hippocampus and in the cortices (as seen in the Human Allen Brain Atlas, http://www.brain-map.org/). Several mouse models have been generated to characterise the properties of the different isoforms and domains. Knockdown models have shown that the long DCLK1 variant is implicated in axogenesis as well as cortical and hippocampal development [18], [19], [20]. Mice which over-express the kinase domain (in the C terminal part of the protein) showed dysregulation of the calmodulin-dependent protein kinase activity, microtubule-associated vesicle transport and GABA-ergic neurotransmission pathways [21]. Subsequently they displayed an increase in anxiety behaviour [22]. Finally in a transgenic mouse model over-expressing *CARP*, there was consolidation of contextual fear memories [23].

The potential role of BDNF in psychiatric disorders has been extensively studied at the gene and protein levels [24], [25], [26], [27], though no clear conclusion has been reached. In this study, we aimed to investigate the effect of genetic variants in *DCLK1* on psychiatric disorders which have cognitive dysfunction as a strong phenotypic component [1], [2], [3], [4], [5], [6], [7]. We chose to screen the entire gene for association, rather than focusing on the genetic variants associated with cognitive traits, to account for possible allelic heterogeneity that could be due to the different samples screened or to the different phenotypes tested. We first mined existing datasets by extracting information from published genome wide association studies (GWAS) of cases with SCZ, BP or ADHD, and then added additional samples that we genotyped ourselves. Considering that many genes have been found to have an effect across several of these psychiatric disorders, and that these disorders probably share a common genetic susceptibility [28], [29], we also performed cross-phenotype analyses for the markers that were shared. We found that SNPs in *DCLK1* were associated with all three disease phenotypes. The strongest effect was seen with a SNP in intron 3, which was very strongly associated with SCZ, and with SCZ and ADHD considered together.

## Methods

All studies were carried out in accordance with the tenets of the Declaration of Helsinki and were approved by the respective local Norwegian, German, Danish and British local research ethical committees; see [30], [31], [32], [33], [34], [35], [36]. Written informed consent was given by all participants and in case of minors by their parents.

We chose to extract the data from existing genome wide association studies (GWASs) for cases of SCZ, BP and ADHD when available. P-values for the region covering *DCLK1* ±10 kb, i.e. chr13: 35,230,790-35,613,514 (NCBI build 36) were extracted from these GWASs. In addition, samples that had not been whole-genome typed were genotyped across the same interval. The genotyping of these samples was performed on different platforms; therefore, different sets of markers have been used in the different studies.

A summary of the samples studied, the number of markers extracted or genotyped, and the platform used is given in Table 1. A description of the marker selection is given below.

**Table 1:** Origin of the samples used either in the GWAS mining, or genotyped in the replication samples.

Phenotype	Sample (reference)	Application	Cases	Controls	No. of markers	Covariate code	Genotyping platform
SCZ	German [30]	GWAS mining	484	1300*	135	2	Illumina 550v3
SCZ	British [34]	GWAS mining	479	2937**	85	12	Affymetrix GC500K
SCZ	Danish [32]	Genotyping	481	826	129	8	Illumina Golden Gate
SCZ	Norwegian [32]	Genotyping	160	269	129	9	Illumina Golden Gate
BP	German [35]	GWAS mining	682	1300*	107	2	Illumina 550v3
BP	British [36]	GWAS mining	1868	2937**	107	12	Affymetrix GC500K
BP	American [38]	GWAS mining	461	563	109	n.a.	Pools Illumina 550
BP	Bosnian/Serbian [35]	Replication	124	115	20	1	Sequenom Massarray
BP	German [35]	Replication	378	768	20	3	Sequenom Massarray
BP	Spanish [35]	Replication	298	400	20	4	Sequenom Massarray
BP	Polish [35]	Replication	446	558	20	5	Sequenom Massarray
BP	Romanian [35]	Replication	237	234	20	6	Sequenom Massarray
BP	Russian [35]	Replication	331	332	20	7	Sequenom Massarray
ADHD	Norwegian [31]	Genotyping	466	515	35	10	Sequenom Massarray
ADHD	German [33]	GWAS mining	495	1300*	17	11	Illumina 660

The number of cases and controls, number of markers mined/genotyped and the genotyping technology is shown. The covariate code takes into consideration the possible effect of country of origin and platform used in the genotypic analysis.

* Same control samples used,

** same control samples used. n.a., not applicable as the sample was not used for the merged analyses.

### SCZ samples

Two GWASs were mined for the *DCLK1* region. The first was a British sample described in O'Donovan et al. [34] of 479 cases with SCZ compared to 2937 controls (the WTCCC control set) genotyped on the Affymetrix 500 CHIP. In this sample the *DCLK1* gene was covered by 85 markers. The second was a German sample of 484 cases with SCZ and 1300 controls genotyped on the Illumina 610 BeadChip [35]. In this sample the *DCLK1* gene was covered by 135 markers.

In addition, 129 tagSNPs covering the *DCLK1* gene were selected and included in a Golden Gate Assay to genotype the Scandinavian Collaboration of Psychiatric Etiology (SCOPE) sample of 481 Danish and 160 Norwegian cases with SCZ and 1088 controls (826 Danish and 262 Norwegian); see Håvik et al. [37] for a description of the assay, marker selection and quality control protocols.

### BP samples

Three BP GWASs were mined for the *DCLK1* region. The first was a British WTCCC set of 1868 cases with BP compared to 2938 controls genotyped on the Affymetrix 500 CHIP [36]. *DCLK1* was covered by 107 markers. The second was a NIMH American sample of 461 cases and 563 controls genotyped using DNA pools with the Illumina 550 BeadChip [38]. *DCLK1* was covered by 109 markers. The third was a German sample (BoMa sample) of 682 cases with BP and 1300 controls [30] genotyped using the Illumina Humanmap 610 CHIPs. *DCLK1* was covered by 107 markers. After mining these GWASs, we carried out a replication study in an additional sample of 1814 cases with BP and 2407 controls (see Table 1 for origin details and Cichon et al. [30] for further description of the sample). Twenty three markers had nominal association (P-value<0.05) with BP in any of the BP GWAS mined. Three markers (rs1750719, rs9546404 and rs9575331) were excluded as they were in strong LD with other markers being typed according to Hapmap data from the CEU sample (CEPH-Utah residents with ancestry from northern and western Europe, http://hapmap.ncbi.nlm.nih.gov/index.html.en [39]); see Figure S1.

### ADHD samples

A sample of 466 Norwegian cases and 515 controls [31] was genotyped for markers covering the *DCLK1* gene. For this sample we chose to genotype the markers (n = 20) that had been selected for the replication study in the BP sample. In addition, considering that a study by Neale et al. [40] had reported a possible association between ADHD (in a TDT [transmission/disequilibrium test] design on 956 trios) and the marker rs1539549 (TDT corrected P-value = 2.9×10^−5^) in intron 5 of the gene [40], we chose to include 11 tagSNPs covering the LD block where this SNP was located (for tagging SNP selection protocol see Le Hellard et al. [41]). Finally, as we did not have information about association between the ADHD phenotype and the markers that had shown association to cognitive traits, we also chose to genotype the 12 markers associated with cognition in our previous study [16]. A total of 43 markers were selected, 10 of which failed at design (4 failed at typing, 4 had Hardy Weinberg Equilibrium P-value<0.01, and 2 had minor allele frequency <0.05).

Later, we extracted genotypes from a GWAS of a sample of cases with ADHD and 1300 controls [33]. This sample consists of 495 young patients with ADHD that were recruited and phenotypically characterized in 8 psychiatric outpatient units in Germany for children and adolescents (Aachen, Cologne, Essen, Marburg, Homburg, Trier, Regensburg, and Würzburg). Patients were included if they were diagnosed with ADHD according to DSM-IV [42]. The ascertainment strategy and inclusion criteria have been described previously [43], [44]. Genome wide genotyping for the patients was performed on Human660W-Quadv1 BeadArrays, and for the controls on HumanHap550v3 BeadArrays (Illumina, San Diego, CA, USA) by the Department of Genomics, Life & Brain Center, University of Bonn, Germany. The same controls were used in multiple analyses (i.e. the 3 German GWASs used the same set of controls; see Table 1).

### Single-sample data analysis

All samples were first analysed separately. The following criteria were used for exclusion of markers: call rate <90%, minor allele frequency <5% in controls, Hardy Weinberg Equilibrium P-value<0.001 in controls. DNA samples which had a call rate <90% were excluded.

The associations were tested using a logistic regression (affected status being the outcome predicted by the genotypes, as implemented in Helix Tree SNP & Variation Software, http://www.goldenhelix.com/SNP_Variation/HelixTree/index.html). The genotypes were coded as D = minor allele and d = major allele, under an additive model DD = 0, Dd = 1 and dd = 2, in order to perform genotypic logistic regression with sex and age as covariates.

### Phenotype-specific merged analyses and cross-phenotype analyses

Phenotype-specific merged analyses (or mega-analyses) were performed on the markers common between samples after quality control. The genotypes of the 16 markers that showed association in any of the mined GWAS and that had been typed in the German BP, SCZ and ADHD GWASs were extracted along with rs10507435, which is associated with cognitive traits [40]. In the SCZ samples, 15 markers were analysed in the German GWAS and the Scandinavian (SCOPE) merged genotypes as rs2051090 failed genotyping in the Scandinavian sample. In the BP samples, the 16 markers that had been typed in the German GWAS were used for the merged analysis of the BP samples (German GWAS and replication sample). For ADHD, four markers (rs10507433, rs1171092, rs1171090 and rs7994174) failed genotyping in the Norwegian sample; thus, 12 markers were used for the merged analysis. For cross-phenotype analyses we used the set of 11 markers that had been genotyped across all the disorders.

In these analyses, considering the low number of markers overlapping between the British samples (genotyped on Affymetrix) only the samples genotyped “in house” or with Illumina CHIPs were included. The analyses with the few overlapping markers are presented in Table S8.

The cross-phenotype analyses were performed using a genotypic logistic regression on an additive model using sex and age as covariates. In addition, in order to control for possible confounding effects of geographical location or genotyping platform we included a correction factor which combines both the origin and platform effects (see Table 1). For example, the German samples that were typed on the same platform for the GWASs had the same Country/Study factor, while the German replication sub-sample had a different index because it was typed on another platform.

Owing to the design of our study, in which we mined or genotyped different sets of markers on different sets of samples depending on availability, it is difficult to apply an appropriate permutation-based analysis or a straightforward Bonferroni correction factor, or a permutation test, as many of the markers tested within the different samples or between the samples are correlated by linkage disequilibrium. Hence, all P-values reported in this study are un-corrected and declared significant at a nominal threshold of P = 0.05. As a guideline to significance, we calculated a Nyholt's SNPSpD gene-based correction. To do this, we downloaded genotypes for the CEU sample from HapMap release 3 (http://hapmap.ncbi.nlm.nih.gov/downloads/index.html.en [39]) covering the whole *DCLK1* genomic region. The gene was covered by a total of 594 markers in the CEU sample. Then, using SNPSpD (superlite version: http://gump.qimr.edu.au/general/daleN/SNPSpDsuperlite/), we calculated that there were 340 effective independent signals across the gene [45], giving a gene-wide significance threshold (required to keep the type I error rate at 5%) of 0.00015. It is not possible to calculate how genetically independent ADHD, BP and SCZ are, but a conservative additional correction for testing 3 phenotypes would then give a study-wide significance threshold of 0.00005 (5×10^−5^).

### Sequencing of conserved regions

Six regions in *DCLK1* were selected for sequencing to identify new genetic variants near the SNP rs7989807. Five of these were regions of high inter-species conservation within 10 kb of rs7989807, identified using the UCSC genome browser (http://genome.ucsc.edu/cgi-bin/hgTrackUi?hgsid=164534183&c=chr1&g=multiz28way). The sixth was the region around the binding site for the REST transcription factor, which is 6.3 kb from rs7989807. Details of the regions selected are presented in Table S9. Primer design and sequencing were performed as described in Le Hellard et al. [46]. Primer sequences are available upon request.

## Results

### Association analyses of single phenotypes

For the SCZ case control studies, we observed association with 5 markers in the GWAS: 4 in the British sample [34] and 1 in the German sample [35] (P-values = 0.0047–0.034; Table S1 and S2). In the Scandinavian SCOPE samples [32], 17 markers showed association (lowest P-value = 8×10^−4^ for rs9545255, see Table S3). For the merged analysis of the German GWAS and the Scandinavian samples, we extracted the genotypes for the 16 markers (where these had been typed) that showed evidence for association with SCZ or BP in any of the mined GWASs. We also extracted genotype data for rs10507435, which is strongly associated with cognitive traits [16] and was typed in the German GWAS. The 15 markers that were typed in both the German GWAS and the Scandinavian sample are shown in Table 2. The evidence for association reached the study-wide significance threshold for the marker rs7989807 (P-value = 3.7×10^−5^, odds ratio 1.40 [95% CI: 1.20–1.63]). This association was mostly driven by the Scandinavian cases (i.e. SCOPE) as the Scandinavian and German controls have the same frequencies. Four additional markers showed stronger association (lower P-value) and greater effect (at the odds ratio level) in the merged analysis (see Table 2).

**Table 2:** Association results for the SCZ cases and control samples.

Marker (position)	Sample	LR with covariates	Minor allele (cases - controls)	Odds ratio (95% CI)
**rs10492555** (35607109)	German	0.646	A (0.15-0.14)	1.05 (0.85–1.29)
	Scand.	0.091	A (0.16-0.15)	1.11 (0.92–1.34)
	German+Scand.	0.246	A (0.16-0.14)	1.09 (0.95–1.26)
**rs7327771** (35577512)	German	0.162	A (0.06-0.05)	1.24 (0.91–1.69)
	Scand.	**0.011** *	A (0.06-0.05)	1.28 (0.95–1.72)
	German+Scand.	**0.033** *	A (0.06-0.05)	1.25 (1.01–1.55)
**rs7994174** (35573018)	German	0.057	A (0.09-0.07)	1.28 (0.99–1.67)
	Scand.	**0.013** *	A (0.09-0.07)	1.36 (1.07–1.75)
	German+Scand.	**0.0021** *	A (0.09-0.07)	1.32 (1.11–1.58)
**rs7989807** (35523089)	German	**0.012** *	T (0.13-0.10)	1.34 (1.07–1.68)
	Scand.	**0.00058** *	T (0.14-0.10)	1.43 (1.16–1.76)
	German+Scand.	**0.000037** *	T (0.14-0.10)	1.40 (1.20–1.63)
**rs12874830** (35470040)	German	0.377	G (0.20-0.19)	1.08 (0.90–1.30)
	Scand.	0.109	G (0.20-0.19)	1.06 (0.90–1.26)
	German+Scand.	0.255	G (0.20-0.19)	1.08 (0.95–1.22)
**rs1171090** (35408728)	German	0.892	A (0.26-0.26)	0.98 (0.83–1.16)
	Scand.	0.226	A (0.27-0.26)	1.05 (0.89–1.22)
	German+Scand.	0.736	A (0.27-0.26)	1.02 (0.91–1.14)
**rs1171092** (35407728)	German	0.874	A (0.26-0.26)	0.98 (0.83–1.16)
	Scand.	0.199	A (0.27-0.25)	1.07 (0.91–1.25)
	German+Scand.	0.626	A (0.26-0.26)	1.02 (0.91–1.15)
**rs7990263** (35359216)	German	0.397	A (0.35-0.34)	1.06 (0.91–1.24)
	Scand.	0.909	A (0.32-0.31)	1.05 (0.91–1.22)
	German+Scand.	0.270	A (0.34-0.33)	1.04 (0.94–1.16)
**rs1750921** (35350069)	German	0.271	T (0.23-0.25)	0.91 (0.76–1.08)
	Scand.	0.791	T (0.24-0.23)	1.04 (0.89–1.22)
	German+Scand.	0.720	T (0.24-0.24)	0.97 (0.86–1.09)
**rs1926452** (35342937)	German	0.990	A (0.16-0.16)	0.99 (0.81–1.22)
	Scand.	0.324	A (0.15-0.14)	1.07 (0.88–1.29)
	German+Scand.	0.587	A (0.15-0.15)	1.03 (0.89–1.18)
**rs10507435** (35338996)	German	0.723	G (0.27-0.27)	1.02 (0.87–1.21)
	Scand.	0.810	G (0.25-0.25)	1.02 (0.87–1.20)
	German+Scand.	0.602	G (0.26-0.26)	1.01 (0.90–1.13)
**rs10507433** (35322698)	German	0.509	T (0.19-0.20)	0.94 (0.78–1.13)
	Scand.	0.733	T (0.18-0.18)	0.98 (0.82–1.17)
	German+Scand.	0.583	T (0.18-0.19)	0.95 (0.83–1.07)
**rs9545424** (35281264)	German	0.250	A (0.13-0.12)	1.13 (0.91–1.41)
	Scand.	**0.029** *	A (0.15-0.12)	1.22 (1.00–1.49)
	German+Scand.	**0.026** *	A (0.14-0.12)	1.18 (1.02–1.37)
**rs7999483** (35251437)	German	0.147	C (0.12-0.10)	1.18 (0.94–1.48)
	Scand.	**0.021** *	C (0.13-0.11)	1.24 (1.01–1.53)
	German+Scand.	**0.013** *	C (0.13-0.11)	1.22 (1.05–1.42)
**rs9545297** (35239668)	German	0.389	G (0.15-0.14)	1.09 (0.89–1.34)
	Scand.	**0.0080** *	G (0.17-0.14)	1.32 (1.09–1.59)
	German+Scand.	**0.0062** *	G (0.16-0.14)	1.22 (1.06–1.40)

Analyses are presented for both the allelic regression and the genotypic regression for which the genotypes were recoded under an additive genotypic model; age, sex and country/study were used as covariates. The German sample consists of 484 cases and 1300 controls. The Scandinavian SCOPE (Scandinavian Collaboration on Psychiatric Etiology) sample (Scand.) consists of 641 cases and 1086 controls and was created by merging the Danish and Norwegian samples, which were shown previously to be similar [32], [41]. The 15 markers that were typed or extracted in the 2 samples are presented (1125 cases and 2386 controls); see Tables S2 and S3 for results from all markers in each sample. In Tables 2, 3, 4, and 5, the position (hg18, NCBI36) of each marker is given below its rsID, the minor alleles and their frequencies in cases and controls are given, together with the odds ratio, the 95% confidence interval and the genotype success (call rate).

* indicates significant P-values (<0.05).

In the three BP GWAS that were mined [30], [36], [38], we observed association with 24 markers: 10 in the German sample, 9 in the WTCCC and 5 in the NIMH sample (P-values = 0.0024–0.049; Table S1 and S4). We selected these 24 markers for a replication study, but excluded 3 markers that were in high LD (r2>0.8 in the CEU HapMap sample) with other markers being typed. Additionally, one marker failed at genotyping. Of the 20 markers analysed in the independent replication samples, two showed association (rs7327771 P-value = 0.0053 and rs7994174 P-value = 0.047; see Table S5), but in the opposite direction to that of GWAS sample. In the merged analysis with the 16 markers extracted from the German GWAS, two markers showed nominal association (rs12874830 and rs7999483, P-value = 0.027 and 0.048, respectively; see Table 3).

**Table 3:** Association results for the BP cases and control samples.

Marker (position)	Sample	LR with covariates	Minor allele (cases - controls)	Odds ratio (95% CI)
**rs10492555** (35607109)	German GWAS	0.717	T (0.15-0.14)	1.03 (0.86–1.24)
	Replication	0.388	T (0.16-0.15)	1.05 (0.94–1.18)
	All	0.351	T (0.16-0.15)	1.05 (0.95–1.16)
**rs7327771** (35577512)	German GWAS	**0.029** *	T (0.07-0.05)	1.35 (1.03–1.76)
	Replication	**0.0053** *	T (0.04-0.06)	0.75 (0.61–0.91)
	All	0.297	T (0.05-0.05)	0.90 (0.77–1.06)
**rs7994174** (35573018)	German GWAS	**0.032** *	T (0.09-0.07)	1.29 (1.02–1.63)
	Replication	**0.047** *	T (0.07-0.08)	0.82 (0.70–0.97)
	All	0.691	T (0.07-0.08)	0.95 (0.83–1.09)
**rs7989807** (35523089)	German GWAS	0.088	A (0.12-0.10)	1.20 (0.97–1.48)
	Replication	0.864	A (0.11-0.11)	1.01 (0.88–1.16)
	All	0.283	A (0.11-0.11)	1.07 (0.96–1.20)
**rs12874830**(35470040)	German GWAS	**0.025** *	C (0.21-0.19)	1.19 (1.02–1.40)
	Replication	0.260	C (0.21-0.20)	1.06 (0.95–1.18)
	All	**0.027** *	C (0.21-0.19)	1.10 (1.01–1.20)
**rs1171090** (35408728)	German GWAS	**0.043** *	C (0.29-0.26)	1.16 (1.00–1.34)
	Replication	0.324	C (0.27-0.27)	0.95 (0.86–1.05)
	All	0.771	C (0.27-0.27)	1.01 (0.93–1.10)
**rs1171092** (35407728)	German GWAS	**0.046** *	C (0.29-0.26)	1.15 (1.00–1.34)
	Replication	0.496	C (0.27-0.27)	0.97 (0.88–1.07)
	All	0.596	C (0.27-0.27)	1.02 (0.94–1.11)
**rs7990263** (35359216)	German GWAS	0.874	T (0.34-0.34)	0.98 (0.86–1.13)
	Replication	0.894	T (0.35-0.35)	0.99 (0.91–1.09)
	All	0.852	T (0.35-0.35)	1.00 (0.92–1.07)
**rs2051090** (35352193)	German GWAS	0.509	T (0.45-0.46)	0.95 (0.84–1.09)
	Replication	0.941	T (0.45-0.45)	1.01 (0.92–1.10)
	All	0.723	T (0.45-0.45)	0.99 (0.92–1.06)
**rs1750921** (35350069)	German GWAS	**0.028** *	T (0.22-0.25)	0.84 (0.72–0.98)
	Replication	0.702	T (0.21-0.21)	1.01 (0.91–1.13)
	All	0.333	T (0.22-0.22)	0.94 (0.86–1.03)
**rs1926452** (35342937)	German GWAS	**0.049** *	A (0.13-0.16)	0.83 (0.69–1.00)
	Replication	0.951	A (0.13-0.13)	1.00 (0.88–1.13)
	All	0.234	A (0.13-0.14)	0.93 (0.84–1.03)
**rs10507435** (35338996)	German GWAS	0.161	C (0.25-0.27)	0.89 (0.77–1.04)
	Replication	0.532	C (0.23-0.24)	0.96 (0.87–1.07)
	All	0.182	C (0.24-0.25)	0.93 (0.85–1.01)
**rs10507433** (35322698)	German GWAS	0.558	A (0.19-0.20)	0.95 (0.80–1.12)
	Replication	0.218	A (0.19-0.18)	1.08 (0.95–1.21)
	All	0.514	A (0.19-0.19)	1.02 (0.93–1.12)
**rs9545424** (35281264)	German GWAS	**0.049** *	T (0.14-0.12)	1.20 (0.99–1.46)
	Replication	0.913	T (0.12-0.12)	0.99 (0.87–1.13)
	All	0.222	T (0.12-0.12)	1.05 (0.94–1.17)
**rs7999483** (35251437)	German GWAS	**0.015** *	G (0.13-0.10)	1.27 (1.04–1.56)
	Replication	0.475	G (0.10-0.10)	1.03 (0.90–1.19)
	All	**0.048** *	G (0.11-0.10)	1.10 (0.98–1.23)
**rs9545297** (35239668)	German GWAS	**0.028** *	C (0.16-0.14)	1.22 (1.02–1.46)
	Replication	0.714	C (0.14-0.14)	1.01 (0.89–1.14)
	All	0.126	C (0.15-0.14)	1.07 (0.97–1.18)

Analyses are presented for both the allelic regression and the genotypic regression for which the genotypes were recoded under an additive genotypic model; age, sex and country/study were used as covariates. The German sample consists of 682 cases and 1300 controls; the replication sample consists of 1814 cases and 2407 controls. The 16 markers that were typed or extracted in the 2 samples are presented (2496 cases and 3707 controls); see Tables S4 and S5 for results from all markers in each sample.

* indicates significant P-values (<0.05).

For the ADHD case control studies, 33 markers were genotyped in a Norwegian sample. No marker showed association after quality control (Table S6). Sixteen markers were extracted from a German GWAS of cases with ADHD and controls [33]. Three markers were nominally significant (P-value = 0.00021–0.011; Table S7). For the merged analysis, four of the extracted markers were not genotyped in the Norwegian sample; thus, 12 markers were analysed. Of these, rs7989807, rs12874830 and rs10507435 showed significant association (P-values of 0.016, 3×10^−4^ and 0.036; ORs: 1.29 (1.09–1.53), 1.25(1.09–1.44) and 0.84 (0.74–0.96) respectively; Table 4). The association reported by Neale et al. [40] between ADHD and rs1539549 (P-value = 1×10^−5^), which is in LD with markers associated to cognitive traits [16], was not replicated in the ADHD samples studied here (see Table 4).

**Table 4:** Association results for the ADHD cases and control samples.

Marker (position)	Sample	LR with covariates	Minor allele (cases - controls)	Odds ratio (95% CI)
**rs10492555** (35607109)	NO ADHD	0.168	A (0.18-0.15)	1.20 (0.94–1.52)
	GE ADHD	0.766	A (0.15-0.14)	1.05 (0.86–1.29)
	NO+GE	0.147	A (0.16-0.14)	1.15 (0.98–1.33)
**rs7327771** (35577512)	NO ADHD	0.497	A (0.07-0.06)	1.13 (0.80–1.60)
	GE ADHD	0.201	A (0.06-0.05)	1.19 (0.88–1.62)
	NO+GE	0.165	A (0.06-0.05)	1.21 (0.97–1.52)
**rs7989807** (35523089)	NO ADHD	0.098	T (0.15-0.12)	1.24 (0.96–1.61)
	GE ADHD	0.085	T (0.12-0.10)	1.22 (0.97–1.53)
	NO+GE	**0.016** *	T (0.13-0.10)	1.29 (1.09–1.53)
**rs12874830** (35470040)	NO ADHD	0.283	G (0.20-0.18)	1.13 (0.90–1.42)
	GE ADHD	**0.00022** *	G (0.24-0.19)	1.38 (1.16–1.65)
	NO+GE	**0.00029** *	G (0.22-0.18)	1.25 (1.09–1.44)
**rs7990263** (35359216)	NO ADHD	0.107	A (0.30-0.33)	0.85 (0.70–1.03)
	GE ADHD	0.714	A (0.35-0.34)	1.02 (0.88–1.20)
	Merged	0.481	A (0.32-0.34)	0.93 (0.83–1.05)
**rs2051090** (35352193)	NO ADHD	0.991	T (0.49-0.49)	0.99 (0.83–1.18)
	GE ADHD	0.556	T (0.46-0.46)	1.01 (0.87–1.17)
	NO+GE	0.848	T (0.48-0.47)	1.03 (0.92–1.15)
**rs1750921** (35350069)	NO ADHD	0.310	T (0.26-0.24)	1.11 (0.91–1.37)
	GE ADHD	0.422	T (0.24-0.25)	0.91 (0.77–1.08)
	NO+GE	0.982	T (0.25-0.25)	0.99 (0.87–1.12)
**rs1926452** (35342937)	NO ADHD	0.786	A (0.14-0.14)	1.01 (0.78–1.30)
	GE ADHD	0.276	A (0.14-0.16)	0.88 (0.72–1.09)
	NO+GE	0.365	A (0.14-0.15)	0.92 (0.79–1.07)
**rs10507435** (35338996)	NO ADHD	0.542	G (0.21-0.23)	0.90 (0.72–1.12)
	GE ADHD	0.077	G (0.24-0.27)	0.86 (0.73–1.02)
	NO+GE	**0.036** *	G (0.23-0.26)	0.84 (0.74–0.96)
**rs9545424** (35281264)	NO ADHD	0.055	A (0.10-0.13)	0.75 (0.57–1.00)
	GE ADHD	0.134	A (0.15-0.12)	1.26 (1.02–1.55)
	NO+GE	0.798	A (0.12-0.12)	1.01 (0.86–1.20)
**rs7999483** (35251437)	NO ADHD	0.189	C (0.10-0.12)	0.82 (0.62–1.09)
	GE ADHD	0.287	C (0.12-0.10)	1.20 (0.96–1.51)
	NO+GE	0.809	C (0.11-0.11)	1.03 (0.87–1.23)
**rs9545297** (35239668)	NO ADHD	0.379	G (0.12-0.13)	0.88 (0.67–1.14)
	GE ADHD	0.185	G (0.16-0.14)	1.20 (0.98–1.47)
	NO+GE	0.521	G (0.14-0.14)	1.04 (0.89–1.22)

Analyses are presented for both the allelic regression and the genotypic regression for which the genotypes were recoded under an additive genotypic model; age, sex and country/study were used as covariates. The Norwegian (NO) sample consists of 466 cases and 515 controls; the German (GE) sample consists of 500 cases and 1300 controls. The 12 markers that were typed or extracted in the 2 samples are presented (966 cases and 1815 controls); see Tables S6 and S7 for results from all markers in each sample.

* indicates significant P-values (<0.05).

### Association analyses across phenotypes

Several studies have shown that psychiatric disorders such as BP and SCZ or BP and ADHD might share common genetic susceptibility [28], [29]. In this study our hypothesis was that *DCLK1* could contribute to shared susceptibility in these disorders on the basis of its effect in cognition. We therefore tested the association across-phenotypes. Given that we had genotypes available for all the samples, we chose to perform mega-analyses, i.e. merging together cases from the different studies in one analysis (we did not look at co-morbidity) using covariates for sex and age and a correction factor combining platform and country of origin (see Methods and Table 1). Given that the British BP and SCZ samples have few markers in common with the other samples, the data from the two British samples are not included in the results reported below or in Tables 2, 3, 4, 5, but are available in Table S1. The set of 16 markers extracted from the German GWASs for SCZ, BP and ADHD was used to perform cross-phenotype analyses. Fifteen of the 16 markers were typed in the SCZ and BP samples (one marker failed in the SCZ Scandinavian sample), and 11 of the 16 markers were typed in the ADHD sample.

**Table 5:** Association analyses across ADHD, SCZ and BP phenotypes.

Marker (position)	Sample	LR with covariates	Minor allele (cases - controls)	Odds ratio (95% CI)
**rs10492555** (35607109)	ADHD+BP	0.153	A (0.16-0.15)	1.06 (0.97–1.16)
	ADHD+SCZ	0.058	A (0.16-0.15)	1.10 (0.99–1.23)
	BP+SCZ	0.167	A (0.16-0.15)	1.05 (0.97–1.15)
	ADHD+BP+SCZ	0.071	A (0.16-0.15)	1.06 (0.98–1.15)
**rs7327771** (35577512)	ADHD+BP	0.616	A (0.05-0.05)	0.96 (0.84–1.10)
	ADHD+SCZ	**0.011** *	A (0.06-0.05)	1.23 (1.04–1.45)
	BP+SCZ	0.918	A (0.05-0.05)	1.00 (0.87–1.14)
	ADHD+BP+SCZ	0.634	A (0.05-0.05)	1.03 (0.91–1.16)
**rs7989807** (35523089)	ADHD+BP	0.052	T (0.12-0.11)	1.10 (1.00–1.22)
	ADHD+SCZ	**0.0000042** *	T (0.13-0.10)	1.32 (1.17–1.49)
	BP+SCZ	**0.0021** *	T (0.12-0.11)	1.15 (1.05–1.27)
	ADHD+BP+SCZ	**0.00026** *	T (0.12-0.11)	1.16 (1.06–1.26)
**rs12874830** (35470040)	ADHD+BP	**0.0011** *	G (0.21-0.19)	1.13 (1.05–1.23)
	ADHD+SCZ	**0.0034** *	G (0.21-0.19)	1.15 (1.04–1.27)
	BP+SCZ	**0.035** *	G (0.21-0.19)	1.08 (1.00–1.17)
	ADHD+BP+SCZ	**0.0027** *	G (0.21-0.19)	1.11 (1.04–1.19)
**rs7990263** (35359216)	ADHD+BP	0.544	A (0.34-0.35)	0.97 (0.91–1.04)
	ADHD+SCZ	0.793	A (0.33-0.33)	1.01 (0.93–1.10)
	BP+SCZ	0.548	A (0.34-0.34)	1.02 (0.95–1.08)
	ADHD+BP+SCZ	0.984	A (0.34-0.34)	1.00 (0.94–1.06)
**rs1750921** (35350069)	ADHD+BP	0.732	T (0.22-0.23)	0.98 (0.91–1.06)
	ADHD+SCZ	0.991	T (0.24-0.24)	0.99 (0.91–1.09)
	BP+SCZ	0.511	T (0.22-0.23)	0.97 (0.9–1.05)
	ADHD+BP+SCZ	0.988	T (0.23-0.23)	0.99 (0.93–1.06)
**rs1926452** (35342937)	ADHD+BP	0.329	A (0.13-0.14)	0.95 (0.87–1.04)
	ADHD+SCZ	0.907	A (0.15-0.15)	0.99 (0.89–1.11)
	BP+SCZ	0.718	A (0.14-0.14)	0.98 (0.90–1.07)
	ADHD+BP+SCZ	0.769	A (0.14-0.14)	0.98 (0.91–1.06)
**rs10507435** (35338996)	ADHD+BP	0.084	G (0.23-0.25)	0.93 (0.86–1.00)
	ADHD+SCZ	0.345	G (0.25-0.25)	0.96 (0.87–1.05)
	BP+SCZ	0.524	G (0.24-0.25)	0.97 (0.91–1.05)
	ADHD+BP+SCZ	0.266	G (0.24-0.25)	0.96 (0.90–1.03)
**rs9545424** (35281264)	ADHD+BP	0.389	A (0.12-0.12)	1.03 (0.94–1.14)
	ADHD+SCZ	0.131	A (0.13-0.12)	1.10 (0.97–1.23)
	BP+SCZ	0.054	A (0.13-0.12)	1.09 (0.99–1.19)
	ADHD+BP+SCZ	0.125	A (0.13-0.12)	1.07 (0.98–1.16)
**rs7999483** (35251437)	ADHD+BP	0.129	C (0.11-0.10)	1.07 (0.97–1.19)
	ADHD+SCZ	0.079	C (0.12-0.11)	1.12 (0.99–1.26)
	BP+SCZ	**0.0071** *	C (0.12-0.10)	1.13 (1.03–1.25)
	ADHD+BP+SCZ	**0.022** *	C (0.11-0.10)	1.10 (1.01–1.21)
**rs9545297** (35239668)	ADHD+BP	0.177	G (0.15-0.14)	1.06 (0.97–1.16)
	ADHD+SCZ	**0.027** *	G (0.15-0.14)	1.13 (1.01–1.27)
	BP+SCZ	**0.0093** *	G (0.15-0.14)	1.11 (1.02–1.21)
	ADHD+BP+SCZ	**0.024** *	G (0.15-0.14)	1.09 (1.01–1.18)

For ADHD+BP, 3462 cases versus 4222 controls were analyzed; for ADHD+SCZ, 3621 cases versus 4793 controls were analyzed; for BP+SCZ, 2092 cases versus 2901 controls were analyzed; for ADHD+BP+SCZ, 4587 cases versus 5308 controls were analyzed. The 11 markers typed in all the samples are presented.

* indicates significant P-values (<0.05).

The overall minimum P-value observed was 4×10^−6^ for the marker rs7989807 (OR: 1.32 [1.17–1.49]) in the ADHD and SCZ merged analysis. Although this P-value fails to reach the accepted genome-wide significance threshold of 5×10-8, it does reach the study-wide significance threshold (see Table 5). The same marker was already strongly associated in the SCZ (merged) sample; it did not reach significance in the ADHD sample alone but it did show an effect in the same direction. The increased evidence of association of this marker (or a genetic variant with which it is in LD) comes from the increased the sample size when ADHD and SCZ are combined.

In addition, different markers in the gene show association with different phenotypes (SCZ, BP or ADHD individually, or in different combinations) suggesting either type I or type II errors or allelic heterogeneity (see Table 5).

In order to test for the effect that can be explained by the association with rs7989807, we performed conditional regression in the different samples using rs7989807 as a covariate (in addition to country/study, gender and age covariates). In the ADHD+BP+SCZ, the BP+ADHD and the ADHD+SCZ analyses, only rs12874830, in intron 3, remained significant (P-values = 0.013, 0.002 and 0.02, respectively; Table 5). Most of the association in these analyses can be attributed to an effect picked up by rs7989807, while the rs12874830 association signal might reflect an additional signal in this region. For BP+SCZ, rs9545297 (in the 3′UTR) and rs7999483 (in intron 19) remained nominally significant (P-values = 0.024 and 0.019, respectively; Table 5), which suggests that there could also be another, weaker, signal of association in this region.

### Screening for additional causative genetic variants in a conserved region around rs7989807

The major signal of association observed in this study is located in intron 3 for SCZ and ADHD combined. Considering that intron 3 is large (164 kb) and that long transcripts of the gene are probably controlled by a CpG-rich intronic promoter (as seen on the UCSC genome browser http://genome.ucsc.edu/cgi-bin/hgGateway), we hypothesised that this intron could harbour regulatory regions controlling the expression of the gene and that the association observed could reflect the effect of other genetic variants (in linkage disequilibrium with rs7989807) in these regulatory regions. We sequenced 5 regions of high inter-species conservation, which potentially contain regulatory elements, located within 10 kb of rs7989807 (Table S9). We also sequenced a region located 6.3 kb from rs7989807 (chr13:35529882-35528777, hg18; Table S9) containing a known binding site for the transcriptional repressor REST, which regulates a large network of neuronal genes [47]. The sequencing was performed on genomic DNA from 12 individuals with SCZ, 4 carrying each of the AA, AG or GG rs7989807 genotypes. We identified 16 variants in these sequences, 10 previously reported in dbSNP and 6 not previously reported (but now submitted; http://www.ncbi.nlm.nih.gov/projects/SNP/). None of the 16 variants was in linkage disequilibrium with rs7989807 (see Table S10); hence none was potentially causative for the association observed.

In addition, we screened eQTL databases: the Genotype Tissue Expression eQTL browser (http://www.ncbi.nlm.nih.gov/gtex/GTEX2/gtex.cgi), the eqtl browser at the University of Chicago (http://eqtl.uchicago.edu/cgi-bin/gbrowse/eqtl/) and seeQTL (http://gbrowse.csbio.unc.edu/cgi-bin/gb2/gbrowse/seeqtl/) [48] for the SNPs associated in our cross-phenotype tests (i.e SNPs with P-value<0.05 in Table 5). None of these SNPs were present in these databases.

## Discussion

**Figure 1:**
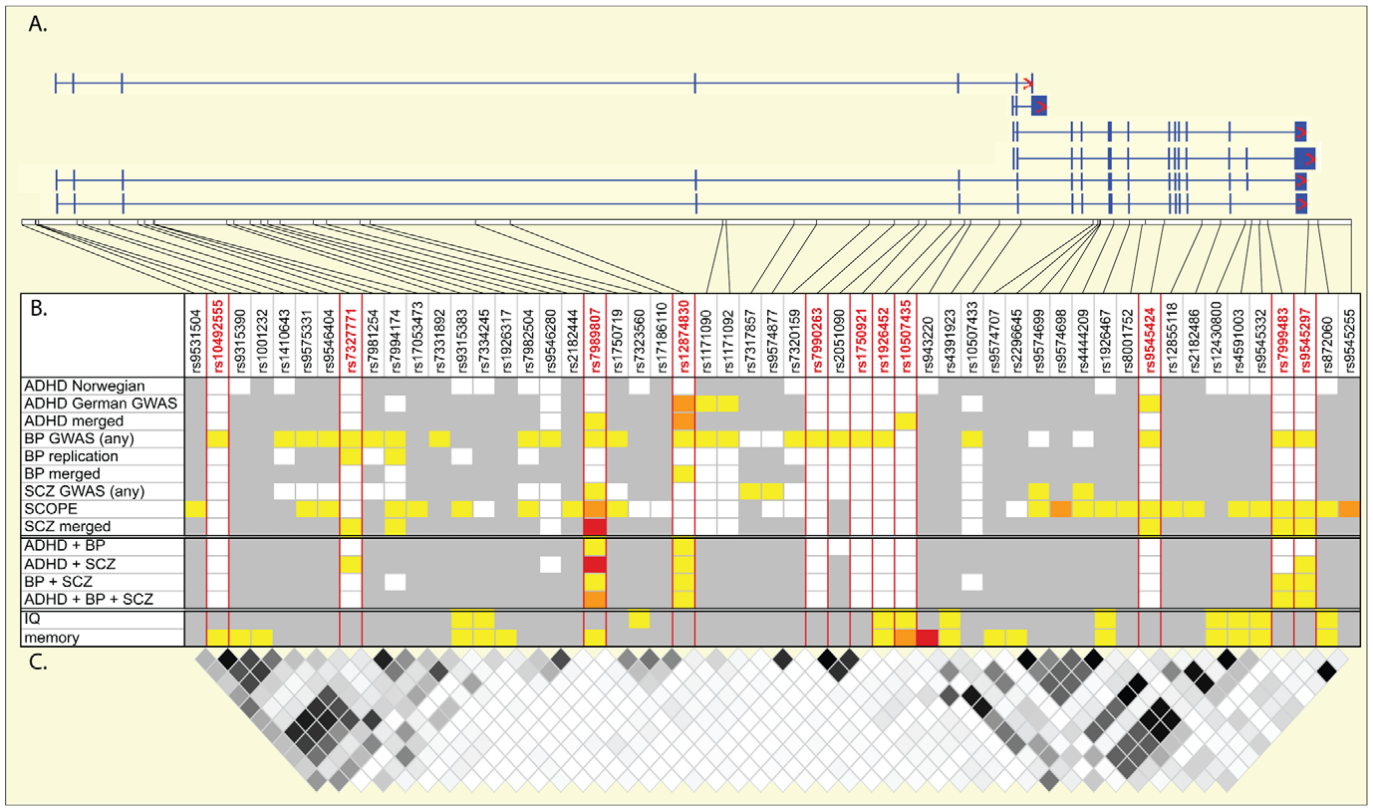
Association of *DCKL1* genetic variants with psychiatric and cognitive traits. Markers are ordered from 5′ to 3′ of the gene, anti-sense to the reference sequence. **A.** Representation of the genomic region covered and of 6 *DCLK1* transcripts (from top to bottom: *DCL*, *CARP*, 2 short variants and 2 long variants). In addition to alternative start sites, the transcripts can be alternatively spliced for part of exon 9, for exon 19 and in the 3′UTR. **B.** All markers showing nominal association to psychiatric traits in this study or to cognitive traits in our previous study [16] are displayed. Color code: yellow, P-value between 0.05 and 0.001; orange, P-value between 0.001 and 0.0001; red, P-value<0.0001; white, P-value>0.05; grey, marker not tested in this sample. The markers used in the cross-phenotype analyses are highlighted in red. **C.** LD between the markers used in the cross-phenotype analyses, and the markers associated with cognitive traits in our previous study [16]. LD is displayed using a r^2^ scale ranging from r^2^ = 1 in black to r^2^ = 0 in white.

In this study we show that genetic variants in *DCLK1* are associated across psychiatric disorders. In our previous study, we demonstrated association across neuropsychological functions [16]. This points to a potential effect of these *DCLK1* variants on central neuronal functions. Figure 1 summarises the results from this study on psychiatric disorders and from our previous studies of association to cognitive traits [16].

In these two studies, we observed association of several markers in the gene with the different phenotypes. It is plausible that several variants in the gene could have an influence on the different phenotypes at different effect sizes. Similar observations of trait-associated allelic heterogeneity have been reported for genes associated with cognition and psychiatric disorders. For instance *DISC1*, which was first reported as a translocated gene segregating with SCZ in a Scottish family [49], has since been associated in several samples with SCZ, BP or with cognitive abilities such as working memory (for review see Chubb et al. [10]). However, the *DISC1* genetic variants that show the strongest associations vary often within and between traits [10], [50]. Hennah et al. [51] have shown that some of the heterogeneity could be diminished by “locking” these analyses on specific markers using conditional regression;, nevertheless, it seems that several genetic variants in *DISC1* are associated at different levels with several traits [10], [50]. Similar allelic heterogeneity for *DCLK1* could explain why some variants in intron 3 seem to be more strongly associated with SCZ and ADHD, while additional variants in the 3′ of the gene show association with BP, and variants in intron 5 are associated with cognitive traits. At present we cannot exclude the possibility that these variations in associated markers are due to type I or type II errors. Overall, when we consider cognitive and psychiatric traits, it seems that there are 3 main regions of association in the gene: i) intron 3, which shows the strongest signal in the SCZ+ADHD cross–phenotype analysis but is also associated with IQ and memory; ii) intron 5/6, which essentially shows association with memory and IQ; iii) intron 19 and the 3′UTR, which show nominal association across psychiatric disorders and IQ and memory. In order to distinguish the true signals in these regions and their association to the different phenotypes, we will need to carry out high-density genotyping (or imputation) of the gene in large samples of individuals, and probably perform alternative analyses such as conditional regression or haplotype analyses. Hopefully, with the release of large imputed datasets as planned by the Psychiatric GWAS consortium [52] for several traits, it will be possible to get a better coverage of the *DLCK1* region.

As shown in Figure 1, the major signal of association observed in this study is located in intron 3 for SCZ and ADHD. Additional signals of association are also observed in introns 4 and 5 and in the 3′UTR. The available information from eQTL databases is rather limited for this region and our attempt to identify potential regulatory variants by sequencing within intron 3 did not identify any convincing candidates. Regulation of the long and short forms of the transcript is likely to be very complex, as shown by their complex pattern of expression in the mouse and human brains (see the Atlas of the Developing Brain: http://www.brainspan.org). It is probable that several regulatory elements or non coding RNAs in the region are involved in this complex regulation. For instance several signals of histone modification are present in intron 3 in the vicinity of rs7989807 (as seen on the UCSC genome browser, http://genome.ucsc.edu), and several micro RNA binding sites are predicted in the 3′ UTR of *DCLK1* (as seen in the Target Scan browser http://www.targetscan.org). In addition, the 5′ exon of an overlapping gene (*MAB21L1)* was recently predicted to be located within intron 3 of *DCLK1*. Thus, it is difficult at this stage to draw conclusions or even speculate on what biological effects could be associated with the genetic variants implicated in the present study. We are currently working on further characterisation of the expression and functions of the *DCLK1* transcripts. It is also interesting to note that a deletion encompassing *DCLK1* and neighbouring genes was reported in a patient suffering from autism and language deficit by Smith et al. in 2002 [53]. This adds to the evidence that genetic variants in this region may be implicated in general susceptibility to mental disorders. However more work is warranted to understand which genes and variants are responsible.

It is now rather well documented that SCZ and BP probably share some genetic susceptibility [54], [55]. Co-morbidity and shared etiological factors have also been reported for ADHD and BP [5]. Though clinical or familial overlap between SCZ and ADHD has not been widely reported, and studies looking at genetic overlap between these disorders are rare, some groups have nevertheless reported co-segregation of these two disorders in families [56], [57]. Recently, several studies looking at copy number variants (CNVs) have shown that ADHD and SCZ do share several rare CNV variants [58], [59], [60]. Our results present for the first time a gene in which common variants show association with SCZ and ADHD and to a lesser extent with BP. SCZ and ADHD are both characterised by severe cognitive deficits, mostly in attention and general cognition, and they both manifest early in development, which is in accordance with an effect of *DCLK1* on neurodevelopment and cognitive phenotypes. Further cross-phenotype studies on large samples from the Psychiatric GWAS Consortium (PGC) may help to identify additional genes showing similar patterns of effects across phenotypes, thus helping us understand how these diagnoses overlap at the genetic and symptom level. It will also be interesting to integrate these data with results from GWASs of cognitive traits.

## Supporting Information

Figure S1 **Selection of markers for replication and genotype extraction from GWASs.** Heatmap of linkage disequilibrium (LD) between the markers showing association with BP or SCZ at the GWAS mining stage, taken from the HapMap CEU sample (http://hapmap.ncbi.nlm.nih.gov) [39], Markers showing association were selected for extraction of genotypes from the German GWAS (when available) and for replication in further samples of BP cases and controls and ADHD cases and controls. When several markers in strong LD (r^2^>0.8) were associated, only one marker was selected for further studies. The LD is displayed using GOLD Heatmap standards for D′ (blue = 0 to red = 1), and the r^2^ values are displayed in the relevant lozenges. In addition the marker rs10507435 was included for its association with cognitive phenotypes [16].
(TIF)

Table S1 **Summary of the data mined in the BP and SCZ GWASs.**
(DOC)

Table S2 **Logistic regression analyses and statistics for the markers extracted from the German GWAS of SCZ.**
(DOC)

Table S3 **Logistic regression analyses and statistics for the markers genotyped in the SCZ Scandinavian sample.**
(DOC)

Table S4 **Logistic regression analyses and statistics for markers extracted from the German (BoMa) GWAS of BP**.
(DOC)

Table S5 **Logistic regression analysis and statistics for the 20 markers genotyped in the BP replication sample.**
(DOC)

Table S6 **Logistic regression analyses and statistics for the 33 markers genotyped in the Norwegian ADHD sample.**
(DOC)

Table S7 **Logistic regression analyses and statistics for the markers extracted from the German ADHD GWAS.**
(DOC)

Table S8 **Results** **across samples including the British GWAS samples for BP (WTCCC) and SCZ (O'Donovan).**
(DOC)

Table S9 **Regions of high inter-species conservation around rs7989807.**
(DOC)

Table S10 **Summary of the genotypes observed for 16 SNPs around rs7989807.**
(DOC)
