# Supplementary figures and images for: DCLK1 Variants Are Associated across Schizophrenia and Attention Deficit/Hyperactivity Disorder
Source: PLoS One. 2012 Apr 23;7(4):e35424. doi: 10.1371/journal.pone.0035424 (PMC3335166; doi:10.1371/journal.pone.0035424)

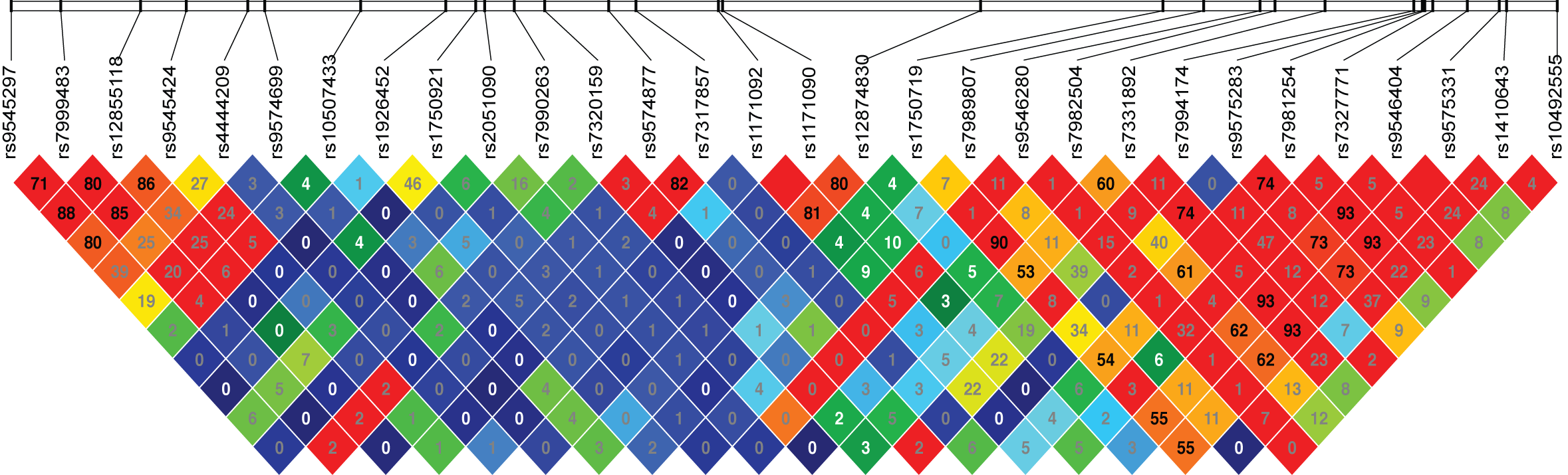

Supplement: Figure S1 — Selection of markers for replication and genotype extraction from GWASs. Heatmap of linkage disequilibrium (LD) between the markers showing association with BP or SCZ at the GWAS mining stage, taken from the HapMap CEU sample (http://hapmap.ncbi.nlm.nih.gov) [39], Markers showing association were selected for extraction of genotypes from the German GWAS (when available) and for replication in further samples of BP cases and controls and ADHD cases and controls. When several markers in strong LD (r2>0.8) were associated, only one marker was selected for further studies. The LD is displayed using GOLD Heatmap standards for D′ (blue = 0 to red = 1), and the r2 values are displayed in the relevant lozenges. In addition the marker rs10507435 was included for its association with cognitive phenotypes [16]. (TIF) [file pone.0035424.s001.tif]
