# Supplementary material for: DCLK1 Variants Are Associated across Schizophrenia and Attention Deficit/Hyperactivity Disorder
Source: PLoS One. 2012 Apr 23;7(4):e35424. doi: 10.1371/journal.pone.0035424 (PMC3335166; doi:10.1371/journal.pone.0035424)
Supplement: Table S1 — Summary of the data mined in the BP and SCZ GWASs. (DOC) [file pone.0035424.s002.doc]

**Table S1. Summary of the data mined in the BP and SCZ GWASs.**

|  |  | **BP GWASs** | | | **SCZ GWASs** | |
| --- | --- | --- | --- | --- | --- | --- |
| **SNP** | **Position** | **German** (29) | **WTCCC** (30) | **American** (32) | **German** (24) | **British** (28) |
| **rs9545297*** | 35,239,668 | **0.028†** | n.t. | > 0.05 | > 0.05 | n.t. |
| **rs7999483*** | 35,251,437 | **0.015†** | n.t. | > 0.05 | > 0.05 | n.t. |
| **rs9545424*** | 35,281,264 | **0.049†** | > 0.05 | > 0.05 | > 0.05 | > 0.05 |
| rs4444209 | 35,295,894 | n.t. | > 0.05 | n.t. | n.t. | **4.7E-03†** |
| rs9574699 | 35,299,909 | n.t. | > 0.05 | n.t. | n.t. | **0.025†** |
| **rs10507433*** | 35,322,698 | > 0.05 | n.t. | **8.7E-03†** | > 0.05 | n.t. |
| **rs1926452*** | 35,342,937 | **0.049†** | > 0.05 | > 0.05 | > 0.05 | > 0.05 |
| **rs1750921*** | 35,350,069 | **0.028†** | > 0.05 | > 0.05 | > 0.05 | > 0.05 |
| **rs2051090*** | 35,352,193 | > 0.05 | **0.021†** | > 0.05 | > 0.05 | > 0.05 |
| **rs7990263*** | 35,359,216 | n.t. | > 0.05 | **0.027†** | > 0.05 | > 0.05 |
| rs7320159 | 35,366,458 | n.t. | **0.045†** | n.t. | n.t. | > 0.05 |
| rs9574877 | 35,381,667 | n.t. | > 0.05 | > 0.05 | n.t. | **0.034†** |
| rs7317857 | 35,388,156 | n.t. | > 0.05 | n.t. | n.t. | **0.012†** |
| **rs1171092*** | 35,407,728 | **0.046†** | n.t. | **0.011†** | > 0.05 | n.t. |
| **rs1171090*** | 35,408,728 | **0.043†** | n.t. | n.t. | > 0.05 | n.t. |
| **rs12874830*** | 35,470,040 | **0.025†** | n.t. | > 0.05 | > 0.05 | n.t. |
| rs1750719 | 35,513,408 | n.t. | **0.022**** | > 0.05 | n.t. | > 0.05 |
| **rs7989807*** | 35,523,089 | > 0.05 | n.t. | **0.016†** | **0.012†** | n.t. |
| rs7982504 | 35,540,023 | n.t. | 0.014† | n.t. | n.t. | > 0.05 |
| rs7331892 | 35,551,923 | n.t. | **8.3E-03†** | n.t. | n.t. | > 0.05 |
| **rs7994174*** | 35,573,018 | **0.032†** | > 0.05 | **2.4E-03†** | > 0.05 | > 0.05 |
| rs7981254 | 35,575,615 | n.t. | **3.0E-03†** | n.t. | n.t. | > 0.05 |
| **rs7327771*** | 35,577,512 | **0.029†** | n.t. | > 0.05 | > 0.05 | n.t. |
| rs9546404 | 35,585,732 | n.t. | **9.7E-03**** | n.t. | n.t. | > 0.05 |
| rs9575331 | 35,593,347 | n.t. | **5.9E-03**** | n.t. | n.t. | > 0.05 |
| rs1410643 | 35,595,063 | n.t. | **0.016†** | n.t. | n.t. | > 0.05 |
| **rs10492555*** | 35,607,109 | n.t. | > 0.05 | **0.048†** | > 0.05 | n.t. |

Two BP and three SCZ GWASs were mined for the region chr13:35,231,123-35,613,464 (*DLCK1* reference sequence +/- 10kb each side, NCBI 36). Only the 28 markers that show association (p-value ≤ 0.05) in any of the GWASs are displayed. (The ADHD GWAS became available at a later stage in the study and was not mined at the p-value level). n.t.: not tested. Note that the difference in markers tested and not tested between the German SCZ and the German BP (BoMa) GWASs comes from variation in the number of markers that passed quality control in the 2 sets. The values shown here are allelic logistic regression as reported by the authors in the original publications. For the German BP GWAS, additional markers were available later for merged analysis due to different quality control criteria. * indicates the 15 markers selected for extracting genotypes from the German samples. One additional marker, rs10507435, that was strongly associated with cognition and genotyped in the German GWAS was also included in the extractions. † indicates the markers selected for replication in the BP replication sample of cases and controls. ** indicates 3 markers that were excluded from genotyping of the BP replication sample and the Norwegian ADHD sample because rs1750719 is in LD with rs7982504 (r2 >0.9 in Hapmap CEU sample), and rs9546404 and rs9575331 are in LD with rs7981254 (Figure S1). Markers are ordered according to the genomic reference sequence (NCBI 36), which is anti-sense to the transcription direction (i.e. 3’ to 5’).
