# Supplementary material for: DCLK1 Variants Are Associated across Schizophrenia and Attention Deficit/Hyperactivity Disorder
Source: PLoS One. 2012 Apr 23;7(4):e35424. doi: 10.1371/journal.pone.0035424 (PMC3335166; doi:10.1371/journal.pone.0035424)
Supplement: Table S2 — Logistic regression analyses and statistics for the markers extracted from the German GWAS of SCZ. (DOC) [file pone.0035424.s003.doc]

**Table S2. Logistic regression analysis and statistics for the markers extracted from the German GWAS of SCZ.**

| **Marker** | **Position** | **LR** | **LR Cov** | **CR** | **MA** | **MAF K** | **MAF C** | **OR** | **OR-L** | **OR-U** |
| --- | --- | --- | --- | --- | --- | --- | --- | --- | --- | --- |
| rs9545297 | 35239668 | 0.3907 | 0.389 | 0.99 | g | 0.15 | 0.14 | 1.09 | 0.89 | 1.34 |
| rs7999483 | 35251437 | 0.1504 | 0.147 | 0.99 | c | 0.12 | 0.1 | 1.18 | 0.94 | 1.48 |
| rs9545424 | 35281264 | 0.2566 | 0.25 | 1 | a | 0.13 | 0.12 | 1.13 | 0.91 | 1.41 |
| rs10507433 | 35322698 | 0.5136 | 0.509 | 1 | t | 0.19 | 0.2 | 0.94 | 0.78 | 1.13 |
| rs10507435 | 35338996 | 0.728 | 0.723 | 1 | g | 0.27 | 0.27 | 1.02 | 0.87 | 1.21 |
| rs1926452 | 35342937 | 0.9849 | 0.99 | 1 | a | 0.16 | 0.16 | 0.99 | 0.81 | 1.22 |
| rs1750921 | 35350069 | 0.2817 | 0.271 | 1 | t | 0.23 | 0.25 | 0.91 | 0.76 | 1.08 |
| rs2051090 | 35352193 | 0.4537 | 0.453 | 0.99 | t | 0.44 | 0.46 | 0.94 | 0.81 | 1.09 |
| rs7990263 | 35359216 | 0.3994 | 0.397 | 0.99 | a | 0.35 | 0.34 | 1.06 | 0.91 | 1.24 |
| rs1171092 | 35407728 | 0.8812 | 0.874 | 0.99 | a | 0.26 | 0.26 | 0.98 | 0.83 | 1.16 |
| rs1171090 | 35408728 | 0.8994 | 0.892 | 0.99 | a | 0.26 | 0.26 | 0.98 | 0.83 | 1.16 |
| rs12874830 | 35470040 | 0.3796 | 0.377 | 1 | g | 0.2 | 0.19 | 1.08 | 0.9 | 1.3 |
| rs7989807 | 35523089 | **0.0112*** | **0.012*** | 0.99 | t | 0.13 | 0.1 | 1.34 | 1.07 | 1.68 |
| rs7994174 | 35573018 | 0.0613 | 0.057 | 1 | a | 0.09 | 0.07 | 1.28 | 0.99 | 1.67 |
| rs7327771 | 35577512 | 0.17 | 0.162 | 0.99 | a | 0.06 | 0.05 | 1.24 | 0.91 | 1.69 |
| rs10492555 | 35607109 | 0.6353 | 0.646 | 0.99 | a | 0.15 | 0.14 | 1.05 | 0.85 | 1.29 |

Data was taken from a published GWAS of 484 SCZ patients and 1300 controls (24). Markers are ordered according to the genomic reference sequence (NCBI 36), anti-sense to the transcription direction. Individual genotypes for the 16 markers localized in the *DCLK1* gene (+/- 10 kb), and showing association in any of the scans mined, were extracted from the GWAS. * indicates significant p-values (< 0.05). LR: Allelic logistic regression. LR cov: P-values are regressed for age and country by platform effect, to take into account the effect of the different geographical origins and the different genotyping platforms used (when appropriate), on an additive (genotypes are coded 0,1,2) model. CR: call rate. MA: minor allele. MAF K: minor allele frequency in cases. MAF C: minor allele frequency in controls. OR: odds ratio for the minor allele. OR-L: 95% confidence interval lower limit. OR-U: 95% confidence interval upper limit. P-values are reported without correction for multiple testing
