# Supplementary material for: DCLK1 Variants Are Associated across Schizophrenia and Attention Deficit/Hyperactivity Disorder
Source: PLoS One. 2012 Apr 23;7(4):e35424. doi: 10.1371/journal.pone.0035424 (PMC3335166; doi:10.1371/journal.pone.0035424)
Supplement: Table S3 — Logistic regression analyses and statistics for the markers genotyped in the SCZ Scandinavian sample. (DOC) [file pone.0035424.s004.doc]

**Table S3. Logistic regression analyses and statistics for the markers genotyped in the SCZ Scandinavian (SCOPE) sample.**

| **Marker** | **Position** | **LR** | **LR cov** | **CR** | **MA** | **MAF K** | **MAF C** | **OR** | **OR-L** | **OR-U** |
| --- | --- | --- | --- | --- | --- | --- | --- | --- | --- | --- |
| rs9545255 | 35227287 | **1.6E-04*** | **8.0E-04*** | 0.99 | C | 0.43 | 0.37 | 1.31 | 1.13 | 1.5 |
| rs872060 | 35236917 | **0.0111*** | **9.6E-03*** | 0.99 | G | 0.18 | 0.15 | 1.26 | 1.05 | 1.51 |
| rs9545297 | 35239668 | **3.4E-03*** | **8.0E-03*** | 0.99 | C | 0.17 | 0.14 | 1.32 | 1.09 | 1.59 |
| rs7999483 | 35251437 | **0.0383*** | **0.0206*** | 1 | C | 0.13 | 0.11 | 1.24 | 1.01 | 1.53 |
| rs9545332 | 35253680 | **0.0108*** | **0.0323*** | 1 | A | 0.2 | 0.17 | 1.25 | 1.05 | 1.49 |
| rs4591003 | 35256050 | **0.0125*** | 0.0694 | 1 | G | 0.5 | 0.46 | 1.19 | 1.03 | 1.36 |
| rs2182486 | 35265082 | **3.5E-03*** | **0.0262*** | 0.99 | G | 0.33 | 0.38 | 0.8 | 0.69 | 0.93 |
| rs12855118 | 35270410 | **0.0245*** | **0.0187*** | 1 | A | 0.15 | 0.12 | 1.25 | 1.03 | 1.52 |
| rs7333096 | 35271256 | 0.8466 | 0.8307 | 0.99 | G | 0.07 | 0.07 | 1.02 | 0.78 | 1.33 |
| rs9545424 | 35281264 | **0.0479*** | **0.0294*** | 0.99 | A | 0.15 | 0.12 | 1.22 | 1 | 1.49 |
| rs17784597 | 35285832 | 0.0501 | 0.1912 | 1 | C | 0.25 | 0.28 | 0.85 | 0.73 | 1 |
| rs8001752 | 35286737 | **0.0374*** | 0.**0468*** | 1 | A | 0.23 | 0.2 | 1.19 | 1.01 | 1.41 |
| rs7986355 | 35287453 | 0.4989 | 0.8353 | 0.99 | G | 0.28 | 0.27 | 1.05 | 0.9 | 1.22 |
| rs1926467 | 35291375 | **0.0249*** | 0.1232 | 0.99 | A | 0.47 | 0.51 | 0.85 | 0.74 | 0.98 |
| rs9545466 | 35292764 | 0.6474 | 0.7069 | 1 | A | 0.07 | 0.08 | 0.94 | 0.73 | 1.21 |
| rs4444209 | 35295894 | **0.0314*** | 0.0665 | 1 | G | 0.26 | 0.23 | 1.19 | 1.01 | 1.39 |
| rs9574698 | 35299746 | **6.9E-04*** | **5.6E-03*** | 1 | G | 0.3 | 0.25 | 1.3 | 1.11 | 1.52 |
| rs9574699 | 35299909 | **0.0245*** | 0.0564 | 0.99 | G | 0.39 | 0.35 | 1.17 | 1.02 | 1.35 |
| rs2296645 | 35300426 | 0.6795 | 0.8946 | 1 | G | 0.47 | 0.47 | 0.97 | 0.84 | 1.11 |
| rs9545532 | 35306524 | 0.6789 | 0.4717 | 0.99 | G | 0.37 | 0.36 | 1.03 | 0.89 | 1.18 |
| rs17180873 | 35312853 | 0.0976 | 0.1617 | 1 | G | 0.04 | 0.05 | 0.76 | 0.54 | 1.05 |
| rs2322827 | 35316933 | 0.3311 | 0.393 | 1 | A | 0.08 | 0.09 | 0.88 | 0.69 | 1.13 |
| rs4941820 | 35317470 | 0.838 | 0.6497 | 0.99 | G | 0.36 | 0.36 | 1.01 | 0.87 | 1.17 |
| rs9574740 | 35319732 | 0.7903 | 0.9094 | 0.99 | G | 0.49 | 0.48 | 1.01 | 0.88 | 1.17 |
| rs10507433 | 35322698 | 0.8504 | 0.7332 | 0.99 | A | 0.18 | 0.18 | 0.98 | 0.82 | 1.17 |
| rs1926466 | 35326595 | 0.6715 | 0.2473 | 0.99 | A | 0.39 | 0.39 | 0.96 | 0.84 | 1.11 |
| rs9574747 | 35329698 | 0.2247 | 0.3275 | 0.99 | A | 0.42 | 0.44 | 0.91 | 0.79 | 1.05 |
| rs4941821 | 35330371 | 0.5027 | 0.4046 | 1 | A | 0.3 | 0.31 | 0.95 | 0.81 | 1.1 |
| rs4943344 | 35331036 | 0.7304 | 0.3292 | 0.99 | G | 0.23 | 0.23 | 0.97 | 0.82 | 1.14 |
| rs10507435 | 35338996 | 0.7163 | 0.8104 | 1 | G | 0.25 | 0.25 | 1.02 | 0.87 | 1.2 |
| rs12100412 | 35339752 | 0.5741 | 0.32 | 0.99 | A | 0.1 | 0.09 | 1.06 | 0.84 | 1.34 |
| rs1926452 | 35342937 | 0.4638 | 0.3235 | 1 | A | 0.15 | 0.14 | 1.07 | 0.88 | 1.29 |
| rs11147591 | 35348397 | 0.6668 | 0.4481 | 0.99 | G | 0.24 | 0.24 | 1.03 | 0.88 | 1.21 |
| rs4943346 | 35348937 | 0.5427 | 0.375 | 0.99 | A | 0.2 | 0.19 | 1.05 | 0.88 | 1.25 |
| rs1539549 | 35349881 | 0.6847 | 0.7443 | 0.99 | A | 0.32 | 0.32 | 1.03 | 0.88 | 1.19 |
| rs1750921 | 35350069 | 0.5857 | 0.7905 | 0.99 | A | 0.24 | 0.23 | 1.04 | 0.89 | 1.22 |
| rs2185868 | 35350141 | 0.9622 | 0.6509 | 0.99 | G | 0.17 | 0.17 | 0.99 | 0.83 | 1.19 |
| rs9545598 | 35353225 | 0.4277 | 0.2731 | 0.99 | A | 0.08 | 0.09 | 0.9 | 0.71 | 1.15 |
| rs10507437 | 35357643 | 0.6247 | 0.9651 | 0.98 | G | 0.1 | 0.09 | 1.05 | 0.83 | 1.33 |
| rs7990263 | 35359216 | 0.47 | 0.9086 | 0.99 | A | 0.32 | 0.31 | 1.05 | 0.91 | 1.22 |
| rs1171065 | 35359822 | 0.1692 | 0.342 | 1 | C | 0.19 | 0.17 | 1.13 | 0.94 | 1.34 |
| rs1171068 | 35360805 | 0.4039 | 0.686 | 1 | A | 0.11 | 0.12 | 0.91 | 0.73 | 1.13 |
| rs1539546 | 35361601 | 0.6931 | 0.8786 | 0.99 | C | 0.4 | 0.39 | 1.02 | 0.89 | 1.18 |
| rs9531153 | 35365044 | 0.8411 | 0.7858 | 0.99 | G | 0.15 | 0.15 | 0.98 | 0.81 | 1.18 |
| rs7320159 | 35366458 | 0.3459 | 0.629 | 0.99 | G | 0.11 | 0.12 | 0.9 | 0.72 | 1.11 |
| rs1343187 | 35371698 | 0.8182 | 0.6776 | 1 | G | 0.26 | 0.27 | 0.98 | 0.84 | 1.14 |
| rs9574877 | 35381667 | 0.8516 | 0.6782 | 0.99 | G | 0.17 | 0.18 | 0.98 | 0.82 | 1.17 |
| rs1171080 | 35385286 | 0.8992 | 0.7985 | 0.99 | A | 0.17 | 0.17 | 0.98 | 0.82 | 1.18 |
| rs1891673 | 35389659 | 0.5651 | 0.6764 | 1 | G | 0.31 | 0.32 | 0.95 | 0.82 | 1.11 |
| rs1171055 | 35395961 | 0.9203 | 0.9217 | 0.99 | A | 0.06 | 0.06 | 0.98 | 0.74 | 1.31 |
| rs7989245 | 35397597 | 0.2631 | 0.156 | 0.99 | G | 0.33 | 0.32 | 1.08 | 0.93 | 1.25 |
| rs1171092 | 35407728 | 0.3934 | 0.1987 | 0.99 | A | 0.27 | 0.25 | 1.07 | 0.91 | 1.25 |
| rs1171090 | 35408728 | 0.5338 | 0.2259 | 0.98 | A | 0.27 | 0.26 | 1.05 | 0.89 | 1.22 |
| rs9545850 | 35414021 | 0.294 | 0.0773 | 0.99 | A | 0.13 | 0.12 | 1.11 | 0.9 | 1.36 |
| rs8000458 | 35419987 | 0.6869 | 0.8615 | 0.99 | A | 0.07 | 0.07 | 1.05 | 0.81 | 1.36 |
| rs1891670 | 35421209 | 0.4435 | 0.7122 | 0.99 | G | 0.13 | 0.12 | 1.08 | 0.88 | 1.33 |
| rs9545925 | 35433192 | 0.2698 | 0.1397 | 0.99 | C | 0.41 | 0.39 | 1.08 | 0.94 | 1.24 |
| rs4943354 | 35450156 | 0.2331 | 0.7671 | 1 | A | 0.05 | 0.06 | 0.83 | 0.62 | 1.12 |
| rs913071 | 35451105 | 0.8678 | 0.3869 | 1 | G | 0.35 | 0.35 | 0.98 | 0.85 | 1.14 |
| rs9546021 | 35458485 | 0.1911 | 0.5366 | 1 | C | 0.17 | 0.15 | 1.13 | 0.94 | 1.36 |
| rs9531314 | 35461010 | 0.5002 | 0.6522 | 0.99 | A | 0.09 | 0.1 | 0.92 | 0.72 | 1.16 |
| rs10507438 | 35464322 | 0.3114 | 0.1382 | 0.99 | C | 0.47 | 0.48 | 0.93 | 0.81 | 1.06 |
| rs9546049 | 35468807 | 0.2257 | 0.4324 | 0.99 | G | 0.08 | 0.09 | 0.86 | 0.67 | 1.09 |
| rs12874830 | 35470040 | 0.4455 | 0.1093 | 0.99 | G | 0.2 | 0.19 | 1.06 | 0.9 | 1.26 |
| rs9546072 | 35473864 | 0.4078 | 0.6238 | 1 | G | 0.1 | 0.11 | 0.91 | 0.72 | 1.13 |
| rs9601892 | 35477647 | 0.616 | 0.9334 | 1 | G | 0.12 | 0.12 | 1.05 | 0.85 | 1.29 |
| rs4941826 | 35479020 | 0.5731 | 0.7744 | 0.99 | A | 0.19 | 0.18 | 1.05 | 0.88 | 1.25 |
| rs1170994 | 35479177 | 0.3279 | 0.2295 | 0.99 | A | 0.17 | 0.18 | 0.91 | 0.76 | 1.09 |
| rs17186110 | 35480061 | 0.2785 | **0.0299*** | 0.99 | C | 0.1 | 0.09 | 1.13 | 0.9 | 1.42 |
| rs12864772 | 35496925 | 0.5112 | 0.2782 | 0.99 | A | 0.32 | 0.3 | 1.05 | 0.9 | 1.21 |
| rs9575162 | 35513122 | 0.0763 | 0.0511 | 0.99 | A | 0.43 | 0.4 | 1.13 | 0.98 | 1.3 |
| rs1750719 | 35513408 | **0.0106*** | 0.0741 | 0.99 | A | 0.33 | 0.38 | 0.82 | 0.71 | 0.95 |
| rs7989807 | 35523089 | **6.4E-04*** | **5-8E-03*** | 0.99 | A | 0.14 | 0.1 | 1.43 | 1.16 | 1.76 |
| rs2182444 | 35526907 | **0.0305*** | **0.0497*** | 1 | G | 0.41 | 0.37 | 1.16 | 1.01 | 1.34 |
| rs7982504 | 35540023 | **0.0161*** | 0.0726 | 1 | A | 0.35 | 0.4 | 0.83 | 0.72 | 0.96 |
| rs17053405 | 35540259 | **8.1E-03*** | 0.0975 | 0.99 | T | 0.08 | 0.05 | 1.44 | 1.1 | 1.89 |
| rs9575231 | 35541022 | 0.881 | 0.4492 | 0.99 | A | 0.08 | 0.08 | 0.98 | 0.76 | 1.25 |
| rs7330329 | 35542724 | 0.7772 | 0.8126 | 1 | A | 0.23 | 0.22 | 1.02 | 0.86 | 1.2 |
| rs9546288 | 35544007 | 0.715 | 0.8691 | 0.99 | G | 0.43 | 0.43 | 1.02 | 0.89 | 1.17 |
| rs7992343 | 35544482 | 0.7942 | 0.818 | 1 | G | 0.43 | 0.43 | 1.01 | 0.88 | 1.17 |
| rs1159388 | 35544706 | **0.0195*** | 0.0849 | 0.99 | C | 0.35 | 0.39 | 0.84 | 0.73 | 0.97 |
| rs7334245 | 35545153 | 0.7475 | 0.8666 | 0.99 | A | 0.43 | 0.43 | 1.02 | 0.88 | 1.17 |
| rs9315383 | 35549855 | **0.0143*** | 0.1767 | 0.99 | C | 0.44 | 0.48 | 0.84 | 0.73 | 0.96 |
| rs9575257 | 35557784 | 0.3036 | 0.9887 | 1 | A | 0.26 | 0.25 | 1.08 | 0.92 | 1.27 |
| rs17053473 | 35572704 | **0.0154*** | **0.0275*** | 1 | A | 0.08 | 0.05 | 1.39 | 1.06 | 1.82 |
| rs7994174 | 35573018 | **0.013*** | **0.0131*** | 0.99 | A | 0.09 | 0.07 | 1.36 | 1.07 | 1.75 |
| rs7327771 | 35577512 | 0.0991 | **0.011*** | 1 | A | 0.06 | 0.05 | 1.28 | 0.95 | 1.72 |
| rs9546404 | 35585732 | **0.0192*** | 0.2466 | 1 | C | 0.46 | 0.5 | 0.84 | 0.73 | 0.97 |
| rs9593801 | 35592402 | **0.0486*** | 0.1129 | 0.99 | G | 0.27 | 0.24 | 1.17 | 1 | 1.37 |
| rs9575331 | 35593347 | **0.0179*** | 0.2345 | 1 | A | 0.46 | 0.5 | 0.84 | 0.73 | 0.97 |
| rs7984436 | 35594255 | 0.1866 | 0.5242 | 0.99 | T | 0.29 | 0.27 | 1.1 | 0.95 | 1.29 |
| rs9602241 | 35600333 | 0.1919 | 0.8778 | 0.99 | A | 0.45 | 0.42 | 1.09 | 0.95 | 1.26 |
| rs10492555 | 35607109 | 0.2414 | 0.0914 | 0.99 | A | 0.16 | 0.15 | 1.11 | 0.92 | 1.34 |
| rs1556060 | 35607477 | 0.9475 | 0.951 | 0.98 | A | 0.12 | 0.12 | 1 | 0.81 | 1.24 |
| rs9531504 | 35610972 | **0.0252*** | **6.2E-03*** | 0.99 | A | 0.2 | 0.23 | 0.82 | 0.69 | 0.97 |
| rs4480666 | 35611946 | 0.5379 | 0.3328 | 1 | G | 0.34 | 0.33 | 1.04 | 0.9 | 1.21 |
| rs7333765 | 35613552 | 0.8227 | 0.798 | 1 | G | 0.17 | 0.18 | 0.97 | 0.81 | 1.17 |
| rs2076799 | 35619182 | 0.5726 | 0.8556 | 1 | G | 0.06 | 0.06 | 1.08 | 0.82 | 1.43 |

Markers were genotyped in the SCZ SCOPE Danish and Norwegian sample (641 cases and 1088 controls) (26). The markers were selected using a tagging SNP strategy (35): genotypes from the CEU population were downloaded from the HapMap and imported into Haploview (52), and aggressive 2-3 marker haplotype tagging SNP selection was performed using the Haploview tagger function (r2 threshold 0.8). In addition, the markers studied in Le Hellard et al.(16) for association to cognition were included. The markers were included in an Illumina Golden Gate Assay and genotyped in-house (31). 129 markers were selected, of which 98 passed the quality controls. * indicates significant p-values (< 0.05). See Table S2 for abbreviations. Markers are ordered according to the genomic reference sequence (NCBI 36). P-values are reported without correction for multiple testing.
