# Supplementary material for: DCLK1 Variants Are Associated across Schizophrenia and Attention Deficit/Hyperactivity Disorder
Source: PLoS One. 2012 Apr 23;7(4):e35424. doi: 10.1371/journal.pone.0035424 (PMC3335166; doi:10.1371/journal.pone.0035424)
Supplement: Table S4 — Logistic regression analyses and statistics for markers extracted from the German (BoMa) GWAS of BP. (DOC) [file pone.0035424.s005.doc]

**Table S4. Logistic regression analyses and statistics for markers extracted from the German (BoMa) GWAS of BP**.

| **Marker** | **Position** | **LR** | **LR Cov** | **CR** | **MA** | **MAF K** | **MAF C** | **OR** | **OR-L** | **OR-U** |
| --- | --- | --- | --- | --- | --- | --- | --- | --- | --- | --- |
| rs9545297 | 35239668 | **0.028*** | **0.028*** | 1 | g | 0.16 | 0.14 | 1.22 | 1.02 | 1.46 |
| rs7999483 | 35251437 | **0.0161*** | **0.015*** | 0.99 | c | 0.13 | 0.1 | 1.27 | 1.04 | 1.56 |
| rs9545424 | 35281264 | 0.0516 | **0.049*** | 1 | a | 0.14 | 0.12 | 1.2 | 0.99 | 1.46 |
| rs10507433 | 35322698 | 0.5559 | 0.558 | 0.99 | t | 0.19 | 0.2 | 0.95 | 0.8 | 1.12 |
| rs10507435 | 35338996 | 0.1563 | 0.161 | 1 | g | 0.25 | 0.27 | 0.89 | 0.77 | 1.04 |
| rs1926452 | 35342937 | 0.0509 | **0.049*** | 1 | a | 0.13 | 0.16 | 0.83 | 0.69 | 1 |
| rs1750921 | 35350069 | **0.0299*** | **0.028*** | 1 | t | 0.22 | 0.25 | 0.84 | 0.72 | 0.98 |
| rs2051090 | 35352193 | 0.5343 | 0.509 | 0.99 | t | 0.45 | 0.46 | 0.95 | 0.84 | 1.09 |
| rs7990263 | 35359216 | 0.8666 | 0.874 | 0.99 | a | 0.34 | 0.34 | 0.98 | 0.86 | 1.13 |
| rs1171092 | 35407728 | **0.0459*** | **0.046*** | 0.99 | a | 0.29 | 0.26 | 1.15 | 1 | 1.34 |
| rs1171090 | 35408728 | **0.0432*** | **0.043*** | 0.99 | a | 0.29 | 0.26 | 1.16 | 1 | 1.34 |
| rs12874830 | 35470040 | **0.028*** | **0.025*** | 1 | g | 0.21 | 0.19 | 1.19 | 1.02 | 1.4 |
| rs7989807 | 35523089 | 0.0801 | 0.088 | 0.99 | t | 0.12 | 0.1 | 1.2 | 0.97 | 1.48 |
| rs7994174 | 35573018 | **0.0307*** | **0.032*** | 1 | a | 0.09 | 0.07 | 1.29 | 1.02 | 1.63 |
| rs7327771 | 35577512 | **0.0296*** | **0.029*** | 0.99 | a | 0.07 | 0.05 | 1.35 | 1.03 | 1.76 |
| rs10492555 | 35607109 | 0.7055 | 0.717 | 0.99 | a | 0.15 | 0.14 | 1.03 | 0.86 | 1.24 |

Data was taken from the BoMa (German) GWAS of 682 BP cases and 1300 controls (29). Individual genotypes for the 16 markers localized in the *DCLK1* gene (+/- 10 kb), and showing association in any of the scans mined, were extracted from the GWAS. * indicates p-values ≤ 0.05. See Table S2 for abbreviations. Markers are ordered according to the genomic reference sequence (NCBI 36). P-values are reported without correction for multiple testing.
