# Supplementary material for: DCLK1 Variants Are Associated across Schizophrenia and Attention Deficit/Hyperactivity Disorder
Source: PLoS One. 2012 Apr 23;7(4):e35424. doi: 10.1371/journal.pone.0035424 (PMC3335166; doi:10.1371/journal.pone.0035424)
Supplement: Table S5 — Logistic regression analysis and statistics for the 20 markers genotyped in the BP replication sample. (DOC) [file pone.0035424.s006.doc]

**Table S5. Logistic regression analysis and statistics for the 20 markers genotyped in the BP replication sample.**

| **Marker** | **Position** | **LR** | **LR Cov** | **CR** | **MA** | **MAF K** | **MAF C** | **OR** | **OR-L** | **OR-U** |
| --- | --- | --- | --- | --- | --- | --- | --- | --- | --- | --- |
| rs9545297 | 35239668 | 0.804 | 0.7144 | 0.99 | C | 0.14 | 0.14 | 1.01 | 0.89 | 1.14 |
| rs7999483 | 35251437 | 0.6028 | 0.4746 | 0.99 | G | 0.1 | 0.1 | 1.03 | 0.9 | 1.19 |
| rs9545424 | 35281264 | 0.9354 | 0.9134 | 0.99 | T | 0.12 | 0.12 | 0.99 | 0.87 | 1.13 |
| rs10507435 | 35338996 | 0.5335 | 0.5322 | 0.97 | C | 0.23 | 0.24 | 0.96 | 0.87 | 1.07 |
| rs1926452 | 35342937 | 0.9797 | 0.9505 | 0.97 | A | 0.13 | 0.13 | 1 | 0.88 | 1.13 |
| rs1750921 | 35350069 | 0.7332 | 0.7024 | 0.97 | T | 0.21 | 0.21 | 1.01 | 0.91 | 1.13 |
| rs2051090 | 35352193 | 0.7794 | 0.9413 | 0.95 | T | 0.45 | 0.45 | 1.01 | 0.92 | 1.1 |
| rs7990263 | 35359216 | 0.994 | 0.8936 | 0.99 | T | 0.35 | 0.35 | 0.99 | 0.91 | 1.09 |
| rs7320159 | 35366458 | 0.3208 | 0.2397 | 0.97 | G | 0.12 | 0.12 | 0.93 | 0.81 | 1.06 |
| rs1171092 | 35407728 | 0.5709 | 0.4957 | 0.97 | A | 0.27 | 0.27 | 0.97 | 0.88 | 1.07 |
| rs1171090 | 35408728 | 0.3766 | 0.3235 | 0.96 | A | 0.27 | 0.27 | 0.95 | 0.86 | 1.05 |
| rs12874830 | 35470040 | 0.2821 | 0.2602 | 0.96 | C | 0.21 | 0.2 | 1.06 | 0.95 | 1.18 |
| rs7989807 | 35523089 | 0.8166 | 0.8641 | 0.95 | A | 0.11 | 0.11 | 1.01 | 0.88 | 1.16 |
| rs7982504 | 35540023 | 0.6657 | 0.7303 | 0.97 | A | 0.39 | 0.4 | 0.98 | 0.89 | 1.07 |
| rs9315383 | 35549855 | 0.7569 | 0.8596 | 0.97 | C | 0.45 | 0.45 | 0.98 | 0.9 | 1.07 |
| rs7994174 | 35573018 | **0.024*** | **0.0474*** | 0.96 | T | 0.07 | 0.08 | 0.82 | 0.7 | 0.97 |
| rs7981254 | 35575615 | 0.8165 | 0.909 | 0.98 | T | 0.45 | 0.45 | 0.98 | 0.9 | 1.07 |
| rs7327771 | 35577512 | **3.5E-03*** | **5.3E-03*** | 0.98 | T | 0.04 | 0.06 | 0.75 | 0.61 | 0.91 |
| rs1410643 | 35595063 | 0.2975 | 0.2676 | 0.99 | T | 0.23 | 0.24 | 0.94 | 0.85 | 1.04 |
| rs10492555 | 35607109 | 0.3552 | 0.3877 | 0.99 | T | 0.16 | 0.15 | 1.05 | 0.94 | 1.18 |

Twenty-one markers showing association (p-value < 0.05) with BP in the mined GWAS were selected for genotyping in a further sample of 2407 controls and 1814 BP cases (24). Markers in complete LD were excluded. Only 20 markers are listed because rs9546280 failed at genotyping. * indicates p-values ≤ 0.05. See Table S2 for abbreviations. Markers are ordered according to the genomic reference sequence (NCBI 36). P-values are reported without correction for multiple testing.
