# Supplementary material for: DCLK1 Variants Are Associated across Schizophrenia and Attention Deficit/Hyperactivity Disorder
Source: PLoS One. 2012 Apr 23;7(4):e35424. doi: 10.1371/journal.pone.0035424 (PMC3335166; doi:10.1371/journal.pone.0035424)
Supplement: Table S6 — Logistic regression analyses and statistics for the 33 markers genotyped in the Norwegian ADHD sample. (DOC) [file pone.0035424.s007.doc]

**Table S6. Logistic regression analyses and statistics for the 33 markers genotyped in the Norwegian ADHD sample.**

| **Marker** | **Position** | **LR** | **LR Cov** | **CR** | **MA** | **MAF K** | **MAF C** | **OR** | **OR-L** | **OR-U** |
| --- | --- | --- | --- | --- | --- | --- | --- | --- | --- | --- |
| rs872060 | 35236917 | 0.1857 | 0.208 | 0.99 | C | 0.13 | 0.15 | 0.84 | 0.65 | 1.08 |
| rs9545297 | 35239668 | 0.343 | 0.379 | 0.99 | G | 0.12 | 0.13 | 0.88 | 0.67 | 1.14 |
| rs7999483 | 35251437 | 0.1841 | 0.189 | 0.99 | C | 0.1 | 0.12 | 0.82 | 0.62 | 1.09 |
| rs9545332 | 35253680 | 0.943 | 0.983 | 0.96 | T | 0.17 | 0.17 | 1 | 0.79 | 1.28 |
| rs4591003 | 35256050 | 0.1085 | 0.134 | 0.98 | C | 0.5 | 0.46 | 1.15 | 0.96 | 1.38 |
| rs12430800 | 35257974 | 0.407 | 0.498 | 0.99 | T | 0.19 | 0.17 | 1.1 | 0.87 | 1.38 |
| rs9545424 | 35281264 | 0.0505 | 0.055 | 0.99 | A | 0.1 | 0.13 | 0.75 | 0.57 | 1 |
| rs1926467 | 35291375 | 0.2328 | 0.257 | 0.99 | A | 0.47 | 0.49 | 0.89 | 0.75 | 1.07 |
| rs4941821 | 35330371 | 0.9131 | 0.874 | 0.99 | T | 0.32 | 0.33 | 0.98 | 0.81 | 1.19 |
| rs4943344 | 35331036 | 0.8398 | 0.682 | 0.99 | C | 0.23 | 0.23 | 1.02 | 0.82 | 1.26 |
| rs943220 | 35337252 | 0.426 | 0.502 | 0.98 | T | 0.38 | 0.39 | 0.92 | 0.77 | 1.11 |
| rs10507434 | 35338404 | 0.9875 | 0.847 | 0.99 | G | 0.14 | 0.14 | 1 | 0.77 | 1.28 |
| rs10507435 | 35338996 | 0.3641 | 0.542 | 0.95 | G | 0.21 | 0.23 | 0.9 | 0.72 | 1.12 |
| rs1926452 | 35342937 | 0.9212 | 0.786 | 1 | A | 0.14 | 0.14 | 1.01 | 0.78 | 1.3 |
| rs11147591 | 35348397 | 0.1037 | 0.073 | 1 | C | 0.25 | 0.22 | 1.18 | 0.96 | 1.46 |
| rs4943346 | 35348937 | 0.7244 | 0.526 | 1 | T | 0.19 | 0.18 | 1.04 | 0.83 | 1.3 |
| rs1539549 | 35349881 | 0.1762 | 0.141 | 0.99 | A | 0.31 | 0.34 | 0.87 | 0.72 | 1.06 |
| rs1539548 | 35349936 | 0.2873 | 0.233 | 0.96 | A | 0.23 | 0.21 | 1.12 | 0.9 | 1.39 |
| rs1750921 | 35350069 | 0.2828 | 0.31 | 0.99 | T | 0.26 | 0.24 | 1.11 | 0.91 | 1.37 |
| rs2051090 | 35352193 | 0.9436 | 0.991 | 1 | T | 0.49 | 0.49 | 0.99 | 0.83 | 1.18 |
| rs7990263 | 35359216 | 0.1106 | 0.107 | 1 | A | 0.3 | 0.33 | 0.85 | 0.7 | 1.03 |
| rs7320159 | 35366458 | 0.4497 | 0.39 | 0.99 | C | 0.1 | 0.11 | 0.89 | 0.67 | 1.19 |
| rs12874830 | 35470040 | 0.2662 | 0.283 | 0.96 | G | 0.2 | 0.18 | 1.13 | 0.9 | 1.42 |
| rs7323560 | 35510465 | 0.0623 | 0.061 | 1 | C | 0.06 | 0.04 | 1.47 | 0.97 | 2.21 |
| rs7989807 | 35523089 | 0.0906 | 0.098 | 1 | T | 0.15 | 0.12 | 1.24 | 0.96 | 1.61 |
| rs7982504 | 35540023 | 0.2229 | 0.301 | 0.99 | A | 0.43 | 0.4 | 1.11 | 0.93 | 1.33 |
| rs7334245 | 35545153 | 0.3751 | 0.431 | 0.98 | T | 0.4 | 0.42 | 0.92 | 0.76 | 1.1 |
| rs9315383 | 35549855 | 0.5436 | 0.593 | 1 | C | 0.49 | 0.47 | 1.05 | 0.88 | 1.26 |
| rs7327771 | 35577512 | 0.4787 | 0.497 | 1 | A | 0.07 | 0.06 | 1.13 | 0.8 | 1.6 |
| rs1410643 | 35595063 | 0.6413 | 0.568 | 0.99 | T | 0.25 | 0.26 | 0.95 | 0.77 | 1.16 |
| rs9315390 | 35607000 | 0.1114 | 0.131 | 0.97 | T | 0.23 | 0.2 | 1.19 | 0.96 | 1.48 |
| rs10492555 | 35607109 | 0.1286 | 0.168 | 0.98 | A | 0.18 | 0.15 | 1.2 | 0.94 | 1.52 |
| rs2296967 | 35642800 | 0.4677 | 0.59 | 0.99 | A | 0.09 | 0.1 | 0.89 | 0.66 | 1.21 |

A total of 466 cases and 511 controls from the Norwegian ADHD sample (25) were analysed using the markers selected for genotyping of the BP sample, together with additional markers associated with cognitive traits. The marker associated with ADHD in the GAIN study (34) is located in a LD block covering introns 4 and 5. Additional markers tagging this block were included in the genotyping of this sample to cover the entire intron 4-5 LD block. See Table S2 for abbreviations. Markers are ordered according to the genomic reference sequence (NCBI 36). P-values are reported without correction for multiple testing.
