# Supplementary material for: DCLK1 Variants Are Associated across Schizophrenia and Attention Deficit/Hyperactivity Disorder
Source: PLoS One. 2012 Apr 23;7(4):e35424. doi: 10.1371/journal.pone.0035424 (PMC3335166; doi:10.1371/journal.pone.0035424)
Supplement: Table S7 — Logistic regression analyses and statistics for the markers extracted from the German ADHD GWAS. (DOC) [file pone.0035424.s008.doc]

**Table S7. Logistic regression analyses and statistics for the markers extracted from the German ADHD GWAS.**

| **Marker** | **LR** | **LR cov** | **CR** | **MA** | **MAF K** | **MAF C** | **OR** | **OR-L** | **OR-U** |
| --- | --- | --- | --- | --- | --- | --- | --- | --- | --- |
| rs9545297 | 0.0688 | 0.1847 | 1 | g | 0.16 | 0.14 | 1.2 | 0.98 | 1.47 |
| rs7999483 | 0.1014 | 0.2868 | 0.99 | c | 0.12 | 0.1 | 1.2 | 0.96 | 1.51 |
| rs9545424 | **0.032*** | 0.1337 | 0.99 | a | 0.15 | 0.12 | 1.26 | 1.02 | 1.55 |
| rs10507433 | 0.2591 | 0.215 | 0.99 | t | 0.18 | 0.2 | 0.89 | 0.74 | 1.08 |
| rs10507435 | 0.0919 | 0.0766 | 0.99 | g | 0.24 | 0.27 | 0.86 | 0.73 | 1.02 |
| rs1926452 | 0.2612 | 0.276 | 0.99 | a | 0.14 | 0.16 | 0.88 | 0.72 | 1.09 |
| rs1750921 | 0.3155 | 0.4218 | 1 | t | 0.24 | 0.25 | 0.91 | 0.77 | 1.08 |
| rs2051090 | 0.8084 | 0.5555 | 0.99 | t | 0.46 | 0.46 | 1.01 | 0.87 | 1.17 |
| rs7990263 | 0.7068 | 0.7136 | 0.99 | a | 0.35 | 0.34 | 1.02 | 0.88 | 1.2 |
| rs1171092 | **0.0232*** | **0.0112*** | 0.99 | a | 0.3 | 0.26 | 1.2 | 1.02 | 1.41 |
| rs1171090 | **0.0232*** | **0.0105*** | 0.99 | a | 0.3 | 0.26 | 1.2 | 1.02 | 1.41 |
| rs12874830 | **2.18E-04*** | **2.16E-04*** | 1 | g | 0.24 | 0.19 | 1.38 | 1.16 | 1.65 |
| rs7989807 | 0.0876 | 0.0845 | 0.99 | t | 0.12 | 0.1 | 1.22 | 0.97 | 1.53 |
| rs7994174 | 0.0696 | 0.0689 | 0.99 | a | 0.09 | 0.07 | 1.27 | 0.98 | 1.65 |
| rs7327771 | 0.253 | 0.2008 | 0.99 | a | 0.06 | 0.05 | 1.19 | 0.88 | 1.62 |
| rs10492555 | 0.6081 | 0.7663 | 0.99 | a | 0.15 | 0.14 | 1.05 | 0.86 | 1.29 |

Markers were extracted from the German GWAS of ADHD (500 cases and 1300 controls) using affected status only as phenotype (27). Individual genotypes for the 16 markers localized in the *DCLK1* gene (+/- 10 kb), and showing association in any of the scans mined, were extracted from the GWAS. * indicates p-values ≤ 0.05. See Table S2 for abbreviations. Markers are ordered according to the genomic reference sequence (NCBI 36). P-values are reported without correction for multiple testing.
