# Supplementary material for: DCLK1 Variants Are Associated across Schizophrenia and Attention Deficit/Hyperactivity Disorder
Source: PLoS One. 2012 Apr 23;7(4):e35424. doi: 10.1371/journal.pone.0035424 (PMC3335166; doi:10.1371/journal.pone.0035424)
Supplement: Table S8 — Results across samples including the British GWAS samples for BP (WTCCC) and SCZ (O'Donovan). (DOC) [file pone.0035424.s009.doc]

**Table S8. Results across samples including the British BP and SCZ samples.**

| **Marker** | **Phenotype** | **LR with covariates** |
| --- | --- | --- |
| rs7990263 | BP | 0.68 |
| rs7990263 | SCZ | 0.64 |
| rs7990263 | Cross phenotypes | 0.34 |
| rs1750921 | BP | 0.21 |
| rs1750921 | SCZ | 0.89 |
| rs1750921 | Cross phenotypes | 0.53 |
| rs1926452 | BP | **0.041*** |
| rs1926452 | SCZ | 0.90 |
| rs1926452 | Cross phenotypes | 0.14 |
| rs9545424 | BP | 0.22 |
| rs9545424 | SCZ | 0.30 |
| rs9545424 | Cross phenotypes | 0.21 |

Owing mainly to a difference in the genotyping platform used, only 4 markers overlapping with all the other samples had been genotyped in the two British samples (O’Donovan et al., ref 28 and WTCCC, ref 30). The covariates used here were sex, age and a country x platform variable (see Methods in main text). For BP, the WTCCC sample, the German BP GWAS sample and the replication sample were used (cases=4494, controls 7382). For SCZ, the O’Donovan sample and the German and SCOPE samples were used (cases= 1604, controls= 6061). For the cross–phenotype analyses, all cases (n= 7063) and all controls, (n= 8984) were included. * indicates p-values ≤ 0.05. See Table S2 for abbreviations. Markers are ordered according to the genomic reference sequence (NCBI 36). P-values are reported without correction for multiple testing; a study-wise Bonferroni correction was calculated as described in the Methods.
