# Supplementary material for: DCLK1 Variants Are Associated across Schizophrenia and Attention Deficit/Hyperactivity Disorder
Source: PLoS One. 2012 Apr 23;7(4):e35424. doi: 10.1371/journal.pone.0035424 (PMC3335166; doi:10.1371/journal.pone.0035424)
Supplement: Table S9 — Regions of high inter-species conservation around rs7989807. (DOC) [file pone.0035424.s010.doc]

**Table S9. Regions of high inter-species conservation around rs7989807.**

| **Screened region** | **SNP ID** | **Position** | **Population** | **D'** | **r^2^** | **LOD** |
| --- | --- | --- | --- | --- | --- | --- |
| **chr13:35529882-35528777** | − | − | − | − | − | − |
| **chr13: 35525644-35523900** | rs2209624 | 35524048 | SCOPE | 0 | 0 | 0 |
|  | rs1926332 | 35524036 | SCOPE | 1 | 0.412 | 2.15 |
|  | ss250607846 | 35524158 | SCOPE | 1 | 0.143 | 1 |
| **chr13: 35523475-35520770** | **rs7989807** | 35523089 | − | − | − | − |
|  | ss250607851 | 35522125 | SCOPE | 1 | 0.043 | 0.31 |
| **chr13: 35519350-35515357** | rs12871873 | 35518844 | CEU | 1 | 0.058 | 2.04 |
|  | rs9531386 | 35518788 | CEU | 0.282 | 0.031 | 0.38 |
|  | rs1926328 | 35516303 | SCOPE | 1 | 0.2 | 0.98 |
|  | rs9315380 | 35515524 | CEU | 0.788 | 0.063 | 1.48 |
|  | ss250607847 | 35515513 | SCOPE | 1 | 0.467 | 1.81 |
|  | rs9593714 | 35515210 | CEU | 1 | 0.001 | 0.05 |
| **chr13: 35514345-35513115** | ss250607848 | 35514197 | SCOPE | 1 | 0.389 | 1.83 |
|  | rs9546227 | 35513953 | CEU | 0.585 | 0.036 | 0.79 |
|  | rs1750719 | 35513408 | CEU | 0.788 | 0.063 | 1.48 |
|  | rs9575162 | 35513122 | CEU | 0.69 | 0.084 | 1.78 |
| **chr13: 35512160-35511137** | ss250607849 | 35511754 | SCOPE | 1 | 0.2 | 0.78 |
|  | ss250607850 | 35511516 | SCOPE | 1 | 0.5 | 2.41 |

Highly conserved regions, as seen on the UCSC genome browser (http://genome.ucsc.edu/cgi-bin/hgGateway, 28-way conservation http://genome.ucsc.edu/cgi-bin/hgTrackUi?hgsid=164534183&c=chr1&g=multiz28way ), were sequenced in 12 affected individuals from the Danish sample with different rs7989807 genotypes (4 AA, 4 AG and 4 GG). The 16 SNPs identified by sequencing are listed. Previously reported SNPs are labeled with their dbSNP rsID; novel SNPs are labeled with their dbSNP sequence submission ID (ssID). Positions are given according to NCBI build 36. Linkage disequilibrium between SNPs identified by sequencing and rs7989807 is given as D’, r^2^ and LOD, and was calculated in Haploview (http://www.broadinstitute.org/haploview/haploview) (52). For the markers available in Hapmap phase II (http://hapmap.ncbi.nlm.nih.gov/downloads/index.html.en) the LD given is based on the CEU population of Utah residents with ancestry from northern and western Europe. For the markers not genotyped in the Hapmap CEU samples we calculated the LD from the 23 SCOPE sequenced individuals. Although the number of individuals sequenced is low for the purposes of calculating LD, the aim was to identify novel SNPs in (almost) complete LD with rs7989807. None of the SNPs identified fulfilled this criterion (as shown here and in Table S10). The SNPs were therefore not followed up further by genotyping the whole sample.
