# Supplementary material for: DCLK1 Variants Are Associated across Schizophrenia and Attention Deficit/Hyperactivity Disorder
Source: PLoS One. 2012 Apr 23;7(4):e35424. doi: 10.1371/journal.pone.0035424 (PMC3335166; doi:10.1371/journal.pone.0035424)
Supplement: Table S10 — Summary of the genotypes observed for 16 SNPs around rs7989807. (DOC) [file pone.0035424.s011.doc]

**Table S10. Summary of the genotypes observed for 16 SNPs around rs7989807.**

|  |  | | | **rs7989807** | | |  | | |
| --- | --- | --- | --- | --- | --- | --- | --- | --- | --- |
|  | **GG** | | | **GA** | | | **AA** | | |
|  | 4 | | | 4 | | | 4 | | |
| **rs2209624** | **AA** | **AG** | **GG** | **AA** | **AG** | **GG** | **AA** | **AG** | **GG** |
|  | 4 | 0 | 0 | 3 | 1 | 0 | 4 | 0 | 0 |
| **rs1926332** | **GG** | **GT** | **TT** | **GG** | **GT** | **TT** | **GG** | **GT** | **TT** |
|  | 4 | 0 | 0 | 2 | 2 | 0 | 0 | 3 | 0 |
| **ss250607846** | **GG** | **GA** | **AA** | **GG** | **GA** | **AA** | **GG** | **GA** | **AA** |
|  | 4 | 0 | 0 | 4 | 0 | 0 | 1 | 3 | 0 |
| **ss250607851** | **GG** | **GA** | **AA** | **GG** | **GA** | **AA** | **GG** | **GA** | **AA** |
|  | 4 | 0 | 0 | 4 | 0 | 0 | 3 | 1 | 0 |
| **rs12871873** | **AA** | **AG** | **GG** | **AA** | **AG** | **GG** | **AA** | **AG** | **GG** |
|  | 2 | 2 | 0 | 1 | 3 | 0 | 4 | 0 | 0 |
| **rs9531386** | **AA** | **AG** | **GG** | **AA** | **AG** | **GG** | **AA** | **AG** | **GG** |
|  | 0 | 0 | 4 | 0 | 2 | 2 | 0 | 0 | 4 |
| **rs1926328** | **GG** | **GT** | **TT** | **GG** | **GT** | **TT** | **GG** | **GT** | **TT** |
|  | 1 | 2 | 1 | 2 | 0 | 0 | 2 | 0 | 0 |
| **rs9315380** | **TT** | **TG** | **GG** | **TT** | **TG** | **GG** | **TT** | **TG** | **GG** |
|  | 1 | 2 | 1 | 2 | 0 | 0 | 2 | 0 | 0 |
| **ss250607847** | **AA** | **AT** | **TT** | **AA** | **AT** | **TT** | **AA** | **AT** | **TT** |
|  | 0 | 2 | 2 | 1 | 1 | 0 | 2 | 0 | 0 |
| **rs9593714** | **CC** | **CA** | **AA** | **CC** | **CA** | **AA** | **CC** | **CA** | **AA** |
|  | 4 | 0 | 0 | 4 | 0 | 0 | 4 | 0 | 0 |
| **ss250607848** | **TT** | **TC** | **CC** | **TT** | **TC** | **CC** | **TT** | **TC** | **CC** |
|  | 3 | 0 | 0 | 2 | 2 | 0 | 0 | 3 | 1 |
| **rs9546227** | **AA** | **AG** | **GG** | **AA** | **AG** | **GG** | **AA** | **AG** | **GG** |
|  | 1 | 1 | 1 | 0 | 1 | 3 | 0 | 0 | 4 |
| **rs1750719** | **AA** | **AC** | **CC** | **AA** | **AC** | **CC** | **AA** | **AC** | **CC** |
|  | 1 | 2 | 1 | 0 | 1 | 3 | 0 | 0 | 4 |
| **rs9575162** | **AA** | **AT** | **TT** | **AA** | **AT** | **TT** | **AA** | **AT** | **TT** |
|  | 0 | 2 | 2 | 3 | 0 | 0 | 4 | 0 | 0 |
| **ss250607849** | **GG** | **GC** | **CC** | **GG** | **GC** | **CC** | **GG** | **GC** | **CC** |
|  | 2 | 2 | 0 | 2 | 2 | 0 | 4 | 0 | 0 |
| **ss250607850** | **GG** | **GA** | **AA** | **GG** | **GA** | **AA** | **GG** | **GA** | **AA** |
|  | 4 | 0 | 0 | 1 | 3 | 0 | 0 | 3 | 1 |

Twelve individuals were analysed, four with each rs7989807 genotype. Results are presented based on the rs7989807 genotype. None of the identified SNPs was specifically associated with rs7989807.
